# Tuning primary shell nitrogen in Fe–N–C for enhanced sulfur redox electrocatalysis

**DOI:** 10.1093/nsr/nwag433

**Published:** 2026-07-13

**Authors:** Yifan Ding, Meng Tian, Shaoqing Chen, Chen Cheng, Jingyuan Zhou, Wenyi Guo, Yu Chen, Miaoyu Lu, Jiaxi Gu, Ziyang Huang, Liang Zhang, Jingyu Sun, Xiangfeng Duan

**Affiliations:** College of Energy, Soochow Institute for Energy and Materials Innovations, Jiangsu Key Laboratory of Advanced Negative Carbon Technologies, Soochow University, Suzhou 215006, China; College of Energy Materials and Chemistry, Inner Mongolia University, Hohhot 010070, China; Interdisciplinary Center for Fundamental and Frontier Sciences, Nanjing University of Science and Technology, Jiangyin 214443, China; College of Energy, Soochow Institute for Energy and Materials Innovations, Jiangsu Key Laboratory of Advanced Negative Carbon Technologies, Soochow University, Suzhou 215006, China; Institute of Functional Nano & Soft Materials, Jiangsu Key Laboratory of Advanced Negative Carbon Technologies, Soochow University, Suzhou 215123, China; Department of Chemistry and Biochemistry, University of California, Los Angeles, CA 90095, USA; Key Laboratory of Advanced Energy Materials Chemistry, Frontiers Science Center for New Organic Matter, College of Chemistry, Nankai University, Tianjin 300071, China; College of Energy, Soochow Institute for Energy and Materials Innovations, Jiangsu Key Laboratory of Advanced Negative Carbon Technologies, Soochow University, Suzhou 215006, China; College of Energy, Soochow Institute for Energy and Materials Innovations, Jiangsu Key Laboratory of Advanced Negative Carbon Technologies, Soochow University, Suzhou 215006, China; College of Energy, Soochow Institute for Energy and Materials Innovations, Jiangsu Key Laboratory of Advanced Negative Carbon Technologies, Soochow University, Suzhou 215006, China; College of Energy, Soochow Institute for Energy and Materials Innovations, Jiangsu Key Laboratory of Advanced Negative Carbon Technologies, Soochow University, Suzhou 215006, China; Institute of Functional Nano & Soft Materials, Jiangsu Key Laboratory of Advanced Negative Carbon Technologies, Soochow University, Suzhou 215123, China; Institute of Functional Nano & Soft Materials, Jiangsu Key Laboratory of Advanced Negative Carbon Technologies, Soochow University, Suzhou 215123, China; College of Energy, Soochow Institute for Energy and Materials Innovations, Jiangsu Key Laboratory of Advanced Negative Carbon Technologies, Soochow University, Suzhou 215006, China; Department of Chemistry and Biochemistry, University of California, Los Angeles, CA 90095, USA; California NanoSystems Institute, University of California, Los Angeles, CA 90095, USA

**Keywords:** lithium-sulfur batteries, Fe–N–C species, single-atom catalysts, primary-shell nitrogen, sulfur redox

## Abstract

Lithium-sulfur (Li-S) batteries promise high energy density but are limited by sluggish sulfur redox kinetics and polysulfide shuttling effect, which lead to poor rate performance and rapid capacity fading. Electrocatalysis has emerged as an effective strategy to accelerate the conversion of polysulfides and improve battery performance. Among various electrocatalysts, metal–nitrogen–carbon catalysts are considered particularly appealing. It is well-recognized that the coordination environment and the type of nitrogen species (e.g. pyridinic or pyrrolic nitrogen) in metal–nitrogen–carbon materials can profoundly influence their catalytic activity. However, the underlying relationship between these different ligand structures and the sulfur redox mechanism remains elusive. Herein, we report a systematic study establishing the relationship between sulfur redox activity and the Fe–N–C sites with precisely regulated primary-shell coordination environments, specifically pyridinic-N (N_pd_) and pyrrolic-N (N_pl_). Comprehensive theoretical simulations and experimental analyses reveal that pyridinic-N coordination exhibits a lower free energy for Li adsorption and positions the *d*-band center of the Fe-N_pd_-single-atom catalyst (SAC) closer to the Fermi level compared to pyrrolic-N. This electronic structure modulation enhances the sulfur redox kinetics. As a result, the optimized pyridinic Fe-N_pd_-SAC electrode delivers favorable electrochemical performance, achieving a specific capacity of 1265 mAh g^−1^ with a capacity fading of <0.05% per cycle. Furthermore, such an electrode enables Li-S full cells with an areal-capacity of 7.1 mAh cm^−2^ at a sulfur loading of 7.2 mg cm^−2^. These findings elucidate the underlying role of first-shell nitrogen coordination in accelerating sulfur redox kinetics, offering insights into the rational construction of practical Li-S batteries.

## Teaser

This work reports underlying roles of first-shell N-coordination of Fe–N–C in expediting sulfur redox kinetics, offering insight into the catalytic essence of single-atomic electrocatalysts in Li–S batteries.

## INTRODUCTION

Rechargeable lithium-sulfur (Li-S) battery has been recognized as a future energy storage technology because of its superior theoretical capacity (1675 mAh g^−1^) and energy density (2600 Wh kg^−1^) [1]. The charge/discharge process in the commonly recognized Li-S system corresponds to the redox process of the active sulfur species in the cathode [2]. Sluggish sulfur redox kinetics and severe lithium polysulfides (LiPSs) shuttling have limited the electrochemical performance and hindered the practical implementation. Hence the development of efficient electrocatalysts for sulfur redox reactions is central [3,4]. Single-atom catalysts (SACs) have emerged as promising candidates for their tunable catalytic centers, maximized exposure of active sites, and exceptional atom utilization efficiency [5]. They have been widely employed to accelerate sulfur redox reactions and enhance electrochemical kinetics. Previous studies have explored various transition metal-based SACs (Fe, Co, Ni, Cu, Zn, Mn) as sulfur hosts and/or separator modifiers over the cathode side to promote sulfur conversion efficiency [6,7]. While these efforts highlight the beneficial role of metal–nitrogen–carbon (M–N–C) SACs in Li-S batteries, challenges remain in fully elucidating the underlying relationship between specific coordination configurations and catalytic performance.

The coordination environments in Fe–N–C electrocatalysts, such as saturated condition (Fe–N_4_) [8], undersaturated condition (Fe–N_2_) [9], or supersaturated condition (Fe–N_5_) [10], play an important role in modulating Li-S catalysis. As such, the Fe–N_4_ SACs with well-defined porphyrin-like structures as the most common type of Fe–N–C catalysts have been explored for Li-S batteries due to their structural simplicity, ease of synthesis and low cost [11]. The Fe metal center in Fe–N_4_ structures, with its partially filled *d*-orbitals, is typically regarded as the primary adsorption site for LiPSs, facilitated by *d–p* orbital hybridization [12]. Tailoring the *d*-band center relative to the Fermi level is a widely adopted strategy to modulate binding energies, enhancing LiPSs adsorption by reducing the occupancy of antibonding states [13]. While the *d–p* orbital interactions between the Fe center and polysulfide anions have been extensively studied in the context of Li-S catalytic mechanisms, the specific role of the coordinating non-metallic nitrogen (N) atoms has received comparatively little attention [14,15]. Notably, the *p*-band centers of these N anions can significantly influence the electron transfer kinetics of the Fe–N_4_ complex by modulating the energy gap between their *p*-orbitals and the Fe *d*-band center. Moreover, these N atoms may form *s–p* orbital hybridizations with Li ions, contributing directly to Li capture and catalytic activity toward LiPSs [16]. Despite these significant promises, the catalytic role of primary-shell N atoms in Fe–N_4_ sites, considering both their electronic influence and structural interactions with the sulfur redox system, remains elusive. Clarifying the function of such coordinating nitrogen in Li-S catalysis is therefore essential for the rational development of high-performance Fe–N_4_ electrocatalysts.

Herein, we present a systematic investigation of Fe–N_4_ SACs sites for Li-S chemistry through combined theoretical and experimental studies. We first performed theoretical simulations of all plausible first-shell nitrogen coordination configurations in Fe–N_4_, elucidating how different N species influence interactions between the central Fe SACs and LiPSs, based upon intrinsic electronic properties and Li adsorption models. The result reveals that pyridinic-N coordination exhibits a lower free energy for Li adsorption and positions the *d*-band center of the Fe–N–C catalyst closer to the Fermi level compared to pyrrolic-N, favoring catalytic polysulfide transformations. Guided by these insights, we achieved selective N coordination via rational precursor design, synthesizing pyridinic-type (Fe-N_pd_-SAC), pyrrolic-type (Fe-N_pl_-SAC), and phthalocyanine-type (Fe-N_pc_-SAC) Fe–N_4_ sites anchored on carbon supports, to elucidate the underlying LiPSs conversion mechanisms as a function of the N-ligand environment. Among them, Fe-N_pd_-SAC exhibits the lowest electronic degeneration and the largest *d*-orbital participation in charge transfer, resulting in enhanced *d*–*p* orbital hybridization and superior catalytic activity. Furthermore, the strongest *s–p* orbital hybridization between the coordinated pyridinic-N and the Li atom in LiPSs not only mitigates the LiPSs shuttling effect but also promotes Li-ion diffusion within the Li-S battery system. As a result, the S/Fe-N_pd_-SAC electrode demonstrates outstanding electrochemical performance, delivering a specific capacity of 1265 mAh g^−1^ at 0.2 C and retaining 95% of its capacity after 100 cycles, and enabling Li-S full cells to deliver an impressive areal-capacity of 7.1 mAh cm^−2^ at a sulfur loading of 7.2 mg cm^−2^.

## RESULTS AND DISCUSSION

### Theoretical evidence of structural and electronic effect

We first carried out density functional theory (DFT) calculations to explore the structure–activity relationship of coordination environments in Fe–N_4_ sites. A series of Fe–N coordination models were constructed to incorporate varying ratios of pyridinic nitrogen (N_pd_) and pyrrolic nitrogen (N_pl_), including FeN_(Pd)4_, FeN_(Pd)3(Pl)1_, FeN_(Pd)2(Pl)2_, FeN_(Pd)1(Pl)3_, FeN_(Pl)4_, and FeN_(Pc)_, with four N_pd_, three N_pd_ with one N_pl_, two N_pd_ with two N_pl_, one N_pd_ with three N_pl_, four N_pl_, and a phthalocyanine-type (N_pc_) coordination, respectively (Fig. 1a). Density of states (DOS) calculations show that all six types of Fe–N_4_ coordination structures exhibit narrow band gaps, suggesting favorable electronic conductivity for efficient electron transfer and electrocatalytic activity (Fig. 1b). Since the type of nitrogen would directly influence the interaction with Li in the polysulfide transformation, we have leveraged the use of Li for investigating and evaluating the catalytic performance of the Fe–N_4_ system. Stripping energy calculations show a clear trend in Li desorption energies, following the order: FeN_(Pd)4_ (1.92 eV) < FeN_(Pd)3(Pl)1_ (1.93 eV) < FeN_(Pd)2(Pl)2_ (2.19 eV) < FeN_(Pd)1(Pl)3_ (2.32 eV) < FeN_(Pl)4_ (2.49 eV) < FeN_(Pc)_ (2.53 eV). This trend suggests that pyridinic-N coordination provides a more favorable environment for Li interaction and desorption, which is critical for efficient LiPSs transformation (Fig. 1c and Fig. S1).

**Figure 1.**
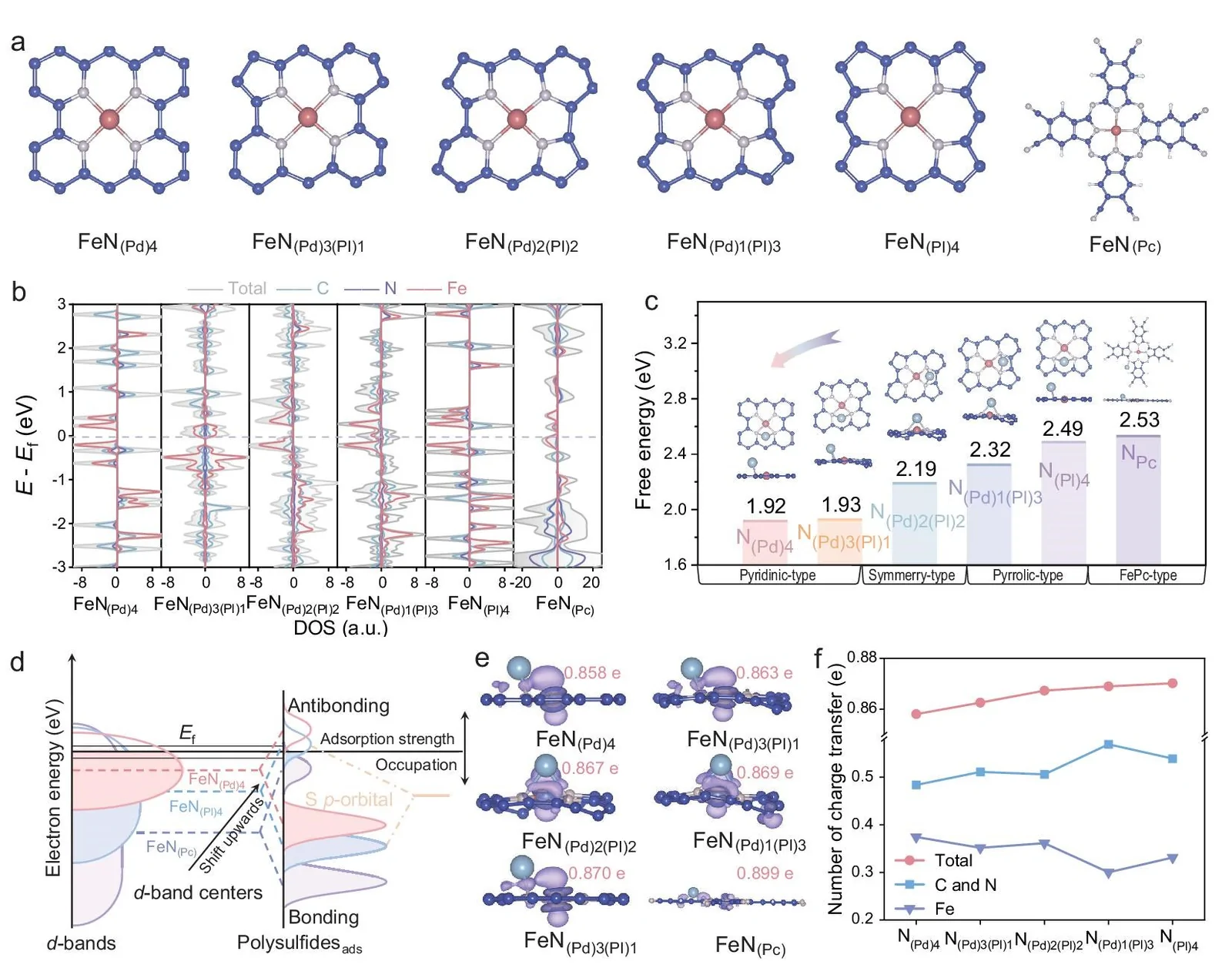
Structural and electronic analysis from DFT calculations. (a) Optimized structures of FeN_(Pd)4_, FeN_(Pd)3(Pl)1_, FeN_(Pd)2(Pl)2_, FeN_(Pd)1(Pl)3_, FeN_(Pl)4_ and FeN_(Pc)_. (b) Density of states of these six compositions. (c) Li adsorption free energy of six compositions. (d) The *d*-band center of FeN_(Pd)4_, FeN_(Pl)4_ and FeN_(Pc)_. (e) Charge density difference for FeN_(Pd)4_, FeN_(Pd)3(Pl)1_, FeN_(Pd)2(Pl)2_, FeN_(Pd)1(Pl)3_, FeN_(Pl)4_ and FeN_(Pc)_. (f) Number of charge transfer for Li adsorption.

We next explore adsorption of polysulfides on different FeN_4_ sites. The bonding and antibonding states are mainly contributed by the hybridization between *p*-orbital of S and *d*-orbital of the transition metal center. According to the valence-bond theory, the occupation of bonding states by electrons can stabilize the structure, whereas occupation of antibonding states destabilizes it. In this regard, the *d*-band center is a critical parameter for assessing adsorption property [17,18]. The antibonding states would be less occupied as the *d*-band center shifts upward, which can enhance the adsorption stability. DFT calculations reveal that the *d*-band center of these three typical catalysts follows the order FeN_(Pd)4_ (−0.950 eV) > FeN_(Pl)4_ (−0.981 eV) > FeN_(Pc)_ (−1.067 eV), indicating Fe-N_pd_-SAC exhibits the strongest adsorption of polysulfides (Fig. 1d and Fig. S2).

We further quantified Bader charge to evaluate the charge transfer between the Li atom and the catalyst. The number of electrons transferred follows the order FeN_(Pd)4_ (0.858 e) < FeN_(Pd)3(Pl)1_ (0.863 e) < FeN_(Pd)2(Pl)2_ (0.867 e) < FeN_(Pd)1(Pl)3_ (0.869 e) < FeN_(Pl)4_ (0.870 e) < FeN_(Pc)_ (0.899 e) (Fig. 1e and Fig. S3). The detailed charge transfer calculations reveal that the number of electron transfer increases as the degree of Fe participation decreases for five catalysts (FeN_(Pd)4_, FeN_(Pd)3(Pl)1_, FeN_(Pd)2(Pl)2_, FeN_(Pd)1(Pl)3_ and FeN_(Pl)4_). Meanwhile, C and N sites make a greater contribution to the charge transfer, demonstrating that the coordinating environment strongly influences the overall electronic structure of the Fe–N_4_ SACs (Fig. 1f). The electron transfer from Li atoms to the Fe–N–C catalyst indicates that its strong affinity could impede Li^+^ mobility (Fig. S4). In this regard, FeN_(Pd)4_ configuration exhibit the lowest electron transfer number and promises the most favorable Li transport performance. These results reveal a fundamental electronic structure principle: the primary-shell N coordination environment governs the orbital hybridization between N 2*p* and metal 3*d* states, which accordingly regulates the *d*-band center, local charge density, and charge-transfer capability of the metal active center. Therefore, our present study readily establishes a generic strategy for M–N–C electrocatalysts: the rational modulation of the *p*-orbital characteristics of first-shell N atoms could be used to tune the electronic structure and catalytic behavior of atomically dispersed metal moieties.

### Identifying Fe–N–C with different coordinated nitrogen

We next prepared three different Fe–N–C SACs by pyrolyzing different structural carbon sources on carbon black (ECP600JD) (Fig. 2a and Fig. S5) [19–21]. In particular, the Fe-N_pd_-SAC was obtained from 2-methylimidazole (C_4_H_6_N_2_) and ferric nitrate nonahydrate (Fe(NO_3_)_3_·9H_2_O) as precursors, the Fe-N_pl_-SAC was obtained by polymerization of 4-dimethylaminopyridine (C_7_H_10_N_2_) and Fe(NO_3_)_3_·9H_2_O, and the Fe-N_pc_-SAC with phthalocyanine microstructure comes from pyrolysis of FePc and dicyandiamide.

**Figure 2.**
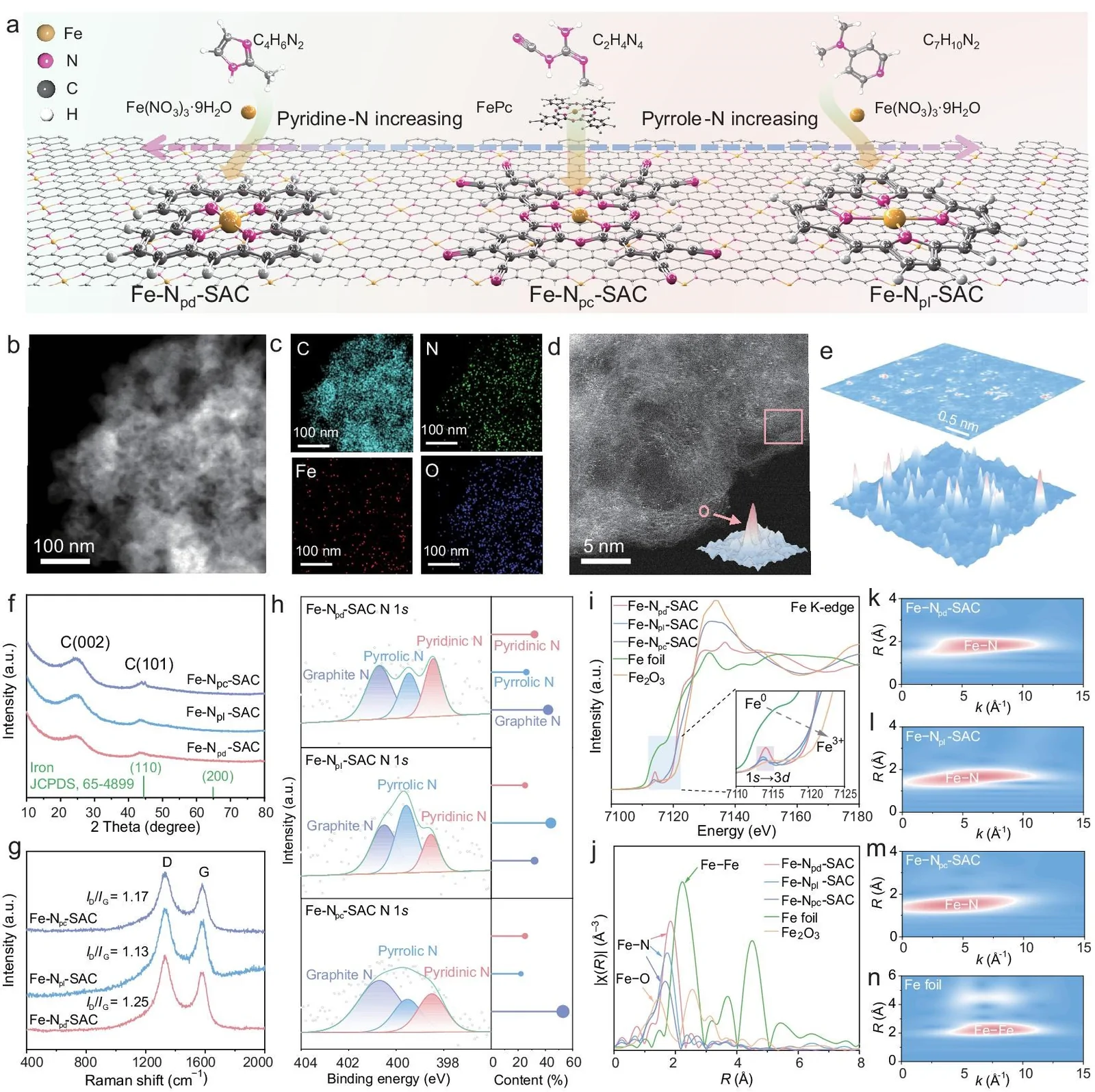
Synthesis and characterization of Fe–N–C catalysts. (a) Preparation essence of Fe-N_pd_-SAC, Fe-N_pl_-SAC and Fe-N_pc_-SAC. (b) STEM image and (c) element maps of Fe-N_pd_-SAC. (d) HAADF-STEM images of Fe-N_pd_-SAC. (e) The local signal intensity profiles labeled with the pink box in (d). (f) XRD patterns and (g) Raman spectra of Fe-N_pd_-SAC, Fe-N_pl_-SAC and Fe-N_pc_-SAC. (h) XPS N 1*s* spectra and N species content of Fe-N_pd_-SAC, Fe-N_pl_-SAC and Fe-N_pc_-SAC. (i) XANES and (j) FT-EXAFS profiles of the Fe-N_pd_-SAC, Fe-N_pl_-SAC, Fe-N_pc_-SAC, Fe foil and Fe_2_O_3_. (k–n) WT contour plots at the Fe K edge of Fe-N_pd_-SAC, Fe-N_pl_-SAC, Fe-N_pc_-SAC and Fe foil.

Scanning electron microscopy (SEM) images show that the Fe-N_pd_-SAC, Fe-N_pl_-SAC and Fe-N_pc_-SAC possess a highly similar porous structure (Fig. S6). Transmission electron microscopy (TEM) studies further confirm similar topography characteristics and internal structures (Fig. S7). Scanning TEM (STEM) image and the energy dispersive X-ray spectroscopy (EDS) maps show that the elements C, N, Fe and O are uniformly distributed in all samples (Fig. 2b and c, and Figs S8 and S9). High-resolution TEM (HRTEM) further revealed the microscopic carbon structure (Fig. S10), suggesting the amorphous nature of carbon support. The high-angle annular dark-field STEM (HAADF-STEM) reveals isolated bright spots corresponding to single Fe atoms (Fig. 2d). Each bright spot exhibits similar signal intensity, indicating that no significant Fe clusters or nanoparticles are generated (Fig. 2e). Moreover, a multitude of single bright spots can be observed in the HAADF-STEM images of Fe-N_pl_-SAC and Fe-N_pc_-SAC, indicating well dispersed single-atoms (Figs S11 and S12).

The X-ray diffraction (XRD) patterns of the three samples showed two broad peaks located at ∼24° and 44° belonging to carbon support, respectively (Fig. 2f). No notable diffraction peaks were found for metallic Fe phase or iron oxide phase, consistent with single atom distribution of Fe atoms. Raman spectra show the characteristic D and G bands of carbon-based material with comparable *I*_D_/*I*_G_ intensity ratios in Fe-N_pd_-SAC, Fe-N_pl_-SAC and Fe-N_pc_-SAC, indicating similar degree of defects in all samples (Fig. 2g). The inductively coupled plasma optical emission spectrometry measurement reveals that Fe metal content reaches *ca.* 0.35 wt% in the Fe-N_pd_-SAC, 0.32 wt% of Fe-N_pl_-SAC and 0.27 wt% of Fe-N_pc_-SAC (Table S1).

X-ray photoelectron spectroscopy (XPS) corroborates the presence of C, N, Fe, and O elements in Fe-N_pd_-SAC, Fe-N_pl_-SAC and Fe-N_pc_-SAC (Fig. S13), with similar C 1*s* spectra (Fig. S14) and O 1*s* spectra (Fig. S15). The N 1*s* spectra (Fig. 2h) show notably different peak intensities at 398.4, 399.5, and 400.6 eV, which are assigned to pyridine-N, pyrrole-N, graphite-N, respectively. The proportions of these three N species varied considerably (Table S2), suggesting distinct N-coordination environments. Previous studies have shown that transition metals tend to coordinate with nitrogen species to form M−N_4_ structures, with pyridinic-N and pyrrolic-N species usually considered to be the main ligands. Among the three samples, the Fe-N_pd_-SAC contained the highest ratio of pyridinic-N species (32%), while Fe-N_pl_-SAC contained the highest ratio of pyrrolic-N species (44%). The Fe 2*p* spectra suggest that Fe in all three samples are in positive oxidation states, indicating no metal cluster formation (Fig. S16). It is worth-noting that the XPS N 1*s* results represent the overall surface nitrogen distribution rather than the exact primary-shell nitrogen coordination environment. As such, the relative contents of pyridinic-, pyrrolic-, and graphitic-N cannot be directly equated with their contributions to Fe–N active sites. The Fe-N_pd_-SAC is more accurately described as having a pyridinic-N-regulated local Fe–N coordination rather than an absolute pyridinic-N-dominated structure. As for the Fe-N_pd_-SAC catalyst, the contribution of other N configurations cannot be completely excluded. Pyrrolic-N may affect local defect structures and polysulfide affinity, while graphitic-N can enhance carbon conductivity and charge delocalization. Therefore, the holistic catalytic performance should be attributed to the synergistic modulation of the carbon matrix and Fe–N sites, with pyridinic-N associated coordination playing a key role.

We further used the X-ray absorption near-edge structure (XANES) and extended X-ray absorption fine structure (EXAFS) analyses to probe the coordination environment and chemical state of Fe and N atoms. The Fe K-edge XANES spectra revealed that the absorption edges for all examined samples are located between Fe foil and Fe_2_O_3_ (Fig. 2i), indicating the oxidation state of Fe between 0 and + 3. The spectra of Fe-N_pd_-SAC, Fe-N_pl_-SAC and Fe-N_pc_-SAC show pre-edge peaks at around 7114 eV ascribed to dipole forbidden 1*s*→3*d* quadrupole transition representing the typical features of Fe–N_4_ configurations (Fig. 2i inset). The Fourier transform (FT) *k*^3^-weighted EXAFS spectra of Fe K-edge show the dominant peak at 1.84, 1.75 and 1.69 Å for Fe-N_pd_-SAC, Fe-N_pl_-SAC and Fe-N_pc_-SAC, corresponding to the first shell of Fe–N scattering path without Fe–Fe (in Fe foil) and Fe–O (in Fe_2_O_3_) interactions, further confirming the isolated Fe atom in the carbon network. Fe-N_pd_-SAC, Fe-N_pl_-SAC and Fe-N_pc_-SAC exhibit a similar Fe coordination environment with four N atoms, whereas Fe-N_pd_-SAC shows the longer Fe–N interatomic distance, attributed to the robust hexagonal ring structure associated with pyridinic-N.

Additionally, the *k*^2^-weighted *k*-space spectra of Fe from EXAFS are fitted (Fig. S17). The wavelet transform (WT) EXAFS of Fe K-edge analysis with powerful resolution in both *k* and *R* spaces was performed to further confirm the isolated dispersion of Fe moiety (Fig. 2k–n). The WT contour plot for Fe-N_pd_-SAC presents a maximum intensity at around 6.5 Å^−1^, corresponding to Fe–N bond configurations, which are at 5.8 and 4.6 Å^−1^ in Fe-N_pl_-SAC and Fe-N_pc_-SAC, respectively. By contrast, the maximum intensity at around 7.8 Å^−1^ in the WT plots of Fe foil is ascribed to the Fe–Fe bond. These XAS analyses confirmed the isolated dispersion of Fe-N_4_-SAC sites in all three coordination structures with similar physicochemical parameters and similar elemental contents. Although the overall distribution of N species manifests merely moderate variation, our XANES/EXAFS results confirm distinct local Fe coordination environments. Therefore, the catalytic behavior is more directly related to the local Fe–N_4_ structure and electronically modulated Fe centers. Thus, the Fe-N_pd_-SAC, Fe-N_pl_-SAC and Fe-N_pc_-SAC sample set offers a robust model system for probing the structure− function relationship between the coordination environment and Li-S chemistry.

### Evaluating sulfur redox kinetics

A series of electrocatalytic-kinetic tests were carried out to investigate the catalytic effect of the three distinct Fe–N_4_ structures on sulfur redox reactions. We have first probed the Li_2_S deposition process with Fe–N_4_ SACs. The integral area of the current response curve at 2.05 V corresponds to the nucleation capacity of Li_2_S (Fig. 3a). Our studies showed that the cell with Fe-N_pd_-SAC showed the highest Li_2_S deposition capacity (392.36 mAh g_s_^−1^) in comparison with that of Fe-N_pl_-SAC (361.76 mAh g_s_^−1^) and Fe-N_pc_-SAC (337.89 mAh g_s_^−1^). The potentiostatic intermittent titration technique (PITT) measurements show similar conclusions (Fig. S18). The SEM images of the dissected cells after potentiostatically discharging at 2.05 V reveal a more uniform Li_2_S deposition on CP with Fe-N_pd_-SAC than that with Fe-N_pl_-SAC and Fe-N_pc_-SAC (Fig. S19) [22].

**Figure 3.**
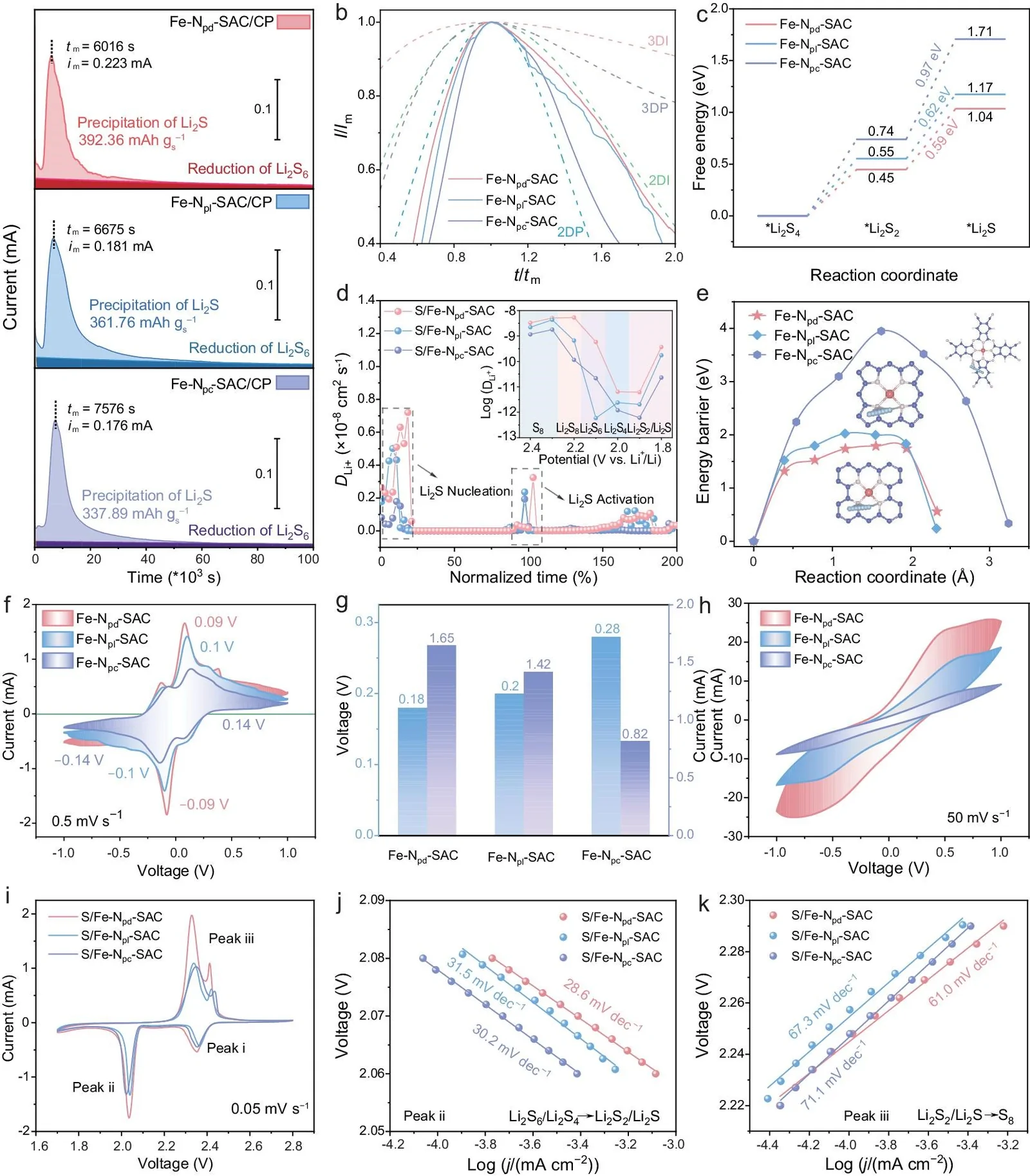
Sulfur redox kinetics tests of Fe–N–C catalysts. (a) Potentiostatic discharge profiles of Li_2_S_8_ solution on Fe-N_4_-SAC samples. (b) Dimensionless current–time transients of different Fe-N_4_-SACs to perform peak fitting according to theoretical 2D and 3D models. (c) Free energy for S species reduction. (d) Calculated diffusion coefficients at different voltages of S/Fe-N_pd_-SAC, S/Fe-N_pl_-SAC and S/Fe-N_pc_-SAC electrodes. (e) Li diffusion energy barriers of different Fe-N_4_-SAC. (f) CV curves of Fe-N_4_-SAC symmetric cells with the addition of Li_2_S_6_ electrolyte at 0.5 mV s^−1^. (g) The potential difference and peak current of Fe-N_4_-SACs. (h) CV curves of Fe-N_4_-SAC symmetric cells at 50 mV s^−1^. (i) Typical CV curves of S/Fe-N_pd_-SAC, S/Fe-N_pl_-SAC and S/Fe-N_pc_-SAC electrodes at 0.05 mV s^−1^. Tafel plots for (j) the liquid–solid reduction process of Li_2_S nucleation and (k) the solid–liquid oxidation process of Li_2_S dissolution.

The cell with Fe-N_pd_-SAC showed the highest peak current (*i*_m_) (0.223 mA) and the shortest nucleation time (*t*_m_) (6016 s) in comparison with those for Fe-N_pl_-SAC (6675 s) and Fe-N_pc_-SAC (7576 s), indicating the fastest Li_2_S nucleation reaction kinetics (Fig. S20). The Li_2_S nucleation rate constant *A* (cm^−2^ s^−1^) and growth rate *k* (cm s^−1^) are related with *t*_m_ as following [23]:


(1) \begin{eqnarray*}
A{k}^2 = 2 \times {\pi}^{ - 1} \times {t}_{\rm m}^{ - 3}.
\end{eqnarray*}

The value of *Ak*^2^ is a criterion of significance reflecting the Li_2_S nucleation and growth process. Importantly, the *Ak*^2^ value of the cell obtained from the potentiostatic profile regarding Fe-N_pd_-SAC is larger than the other two catalysts (Fig. S21), confirming its excellent catalytic activity. Theoretically, Li_2_S deposition follows one of the four modes: the progressive nucleation lies in the separation of nucleation rate and growth rate, leading to non-uniform size distribution of nuclei. The instantaneous nucleation otherwise features synchronized nucleation and growth, resulting in homogeneous dimensions of Li_2_S precipitates. As shown in Fig. 3b, compared with Fe-N_pl_-SAC and Fe-N_pc_-SAC, the nucleation pattern of Fe-N_pd_-SAC is closer to the two-dimensional instantaneous nucleation (2DI) pattern, which implies a more homogeneous Li_2_S deposition process. We further calculated the Gibbs free energy change (Δ*G*) of Li_2_S_4_/Li_2_S_2_ and Li_2_S_2_/Li_2_S conversion (Figs S22–S24), and found that Δ*G* on Fe-N_pd_-SAC is both significantly lower than that on Fe-N_pl_-SAC and Fe-N_pc_-SAC (Fig. 3c), indicating that the Li_2_S nucleation process can be promoted by Fe-N_pd_-SAC.

A galvanostatic intermittent titration technique (GITT) was used to calculate the internal resistances and chemical diffusion coefficient of cells by the dip depth in the discharging and charging profiles (Fig. S25). The polarization during discharging and charging processes can be quantified based on the following equation:


(2) \begin{eqnarray*}
\Delta {R}_{{\rm internal}} = \left| {\Delta {V}_{{\rm QOCV} - {\rm CCV}}} \right|/{I}_{{\rm applied}},
\end{eqnarray*}

where Δ*V*_QOCV–CCV_ is the voltage difference between the points of quasi open-circuit voltage (QOCV) and closed-circuit voltage (CCV), and *I*_applied_ is the applied current [24]. With the same *I*_applied_ value, smaller value of Δ*V*_QOCV–CCV_ means lower Δ*R*_internal_. Importantly, our studies demonstrate that the Fe-N_pd_-SAC electrode exhibits the lowest Δ*R*_internal_ values at the Li_2_S nucleation and activation point in comparison with the S/Fe-N_pl_-SAC and S/Fe-N_pc_-SAC electrodes, consistent with its superior redox kinetics (Fig. S26).

The diffusion coefficient of Li^+^ (*D*_Li^+^_) was further estimated from the GITT results using Fick’s second law [25]:


(3) \begin{eqnarray*}
D = \frac{4}{{\pi \tau }}{\left( {\frac{{{m}_B{V}_M}}{{{M}_BS}}} \right)}^2{\left( {\frac{{{\mathrm{\Delta }}{E}_S}}{{{\mathrm{\Delta }}{E}_\tau }}} \right)}^2,
\end{eqnarray*}

where *τ* is the duration of the current pulse, *m*_B_ and *M*_B_ are the mass and the molecular weight, *V*_m_ is the molar volume of the electrode materials, *S* is the contact area between electrode and electrolyte, Δ*E*_S_ is the change in the steady-state voltage at the end of the relaxation period over a single galvanostatic titration and Δ*E*_τ_ is the total change of cell voltage during a constant-current pulse. The cell with Fe-N_pd_-SAC showed the highest Li^+^ diffusion coefficient (3.24 × 10^−9^ vs. 2.36 × 10^−9^ for Fe-N_pl_-SAC and 1.93 × 10^−9^ cm^2^ s^−1^ for Fe-N_pc_-SAC) (Fig. 3d). In addition, we calculated the Log (*D*_Li^+^_) corresponding to the Li-S battery discharge reduction process and found the optimal reaction kinetics for the Fe-N_pd_-SAC electrode, which can be attributed to faster Li^+^ transport kinetics with pyridinic-N (Fig. 3d inset). Since the Li^+^ diffusion barrier is significant for the kinetic properties, climbing-image nudged elastic band (CINEB) calculations were performed for three typical catalysts (FeN_(Pd)4_, FeN_(Pl)4_ and FeN_(Pc)_). The diffusion barrier follows the order FeN_(Pd)4_ (1.79 eV) < FeN_(Pl)4_ (2.02 eV) < FeN_(Pc)_ (3.95 eV), suggesting FeN_(Pd)4_ exhibits the fastest kinetics for Li^+^ diffusion (Fig. 3e and Fig. S27).

Cyclic voltammetry (CV) tests of symmetrical cells in a Li_2_S_6_ electrolyte at a scan rate of 0.5 mV s^−1^ were performed to explore the catalytic effect for LiPSs conversion (Fig. 3f). Fe-N_pd_-SAC has the smallest potential difference (0.18 mV) and largest peak current (1.65 mA), demonstrating its superior catalytic activity in comparison with the Fe-N_pl_-SAC and Fe-N_pc_-SAC (Fig. 3g). Likewise, a highest redox current was also observed on Fe-N_pd_-SAC at a scan rate of 50 mV s^−1^ (Fig. 3h). At such a high rate, the CV curves of the cell with Fe-N_pd_-SAC electrode showed more obvious redox peaks than those with Fe-N_pl_-SAC and Fe-N_pc_-SAC electrodes, manifesting the excellent catalysis role of Fe-N_pd_-SAC for sulfur transformation.

We further evaluated the electrochemical behavior of Li-S coin-cells with Fe-N_pd_-SAC, Fe-N_pl_-SAC and Fe-N_pc_-SAC. The CV curves at a scan rate of 0.05 mV s^−1^ show two characteristic cathodic peaks appearing at ∼2.3–2.4 V (peak i) and ∼1.9–2.1 V (peak ii), corresponding to the formation of soluble LiPSs and insoluble Li_2_S, respectively (Fig. 3i). The anodic peak in the range of ∼2.3–2.6 V (peak iii) corresponds to the oxidation of Li_2_S during the charging process. The Fe-N_pd_-SAC electrode shows a larger current response and lower voltage polarization than its counterparts, consistent with its superior electrocatalytic activity.

The Tafel slopes of reduction (from Li_2_S*_n_* to Li_2_S) and oxidation (from Li_2_S to S_8_) were calculated to quantify the electrocatalytic effect. The Fe-N_pd_-SAC exhibited the smallest Tafel slope for the sulfur reduction reaction (SRR) (28.6 mV dec^−1^) (Fig. 3j) and the sulfur evolution reaction (SER) (61.0 mV dec^−1^) (Fig. 3k) in comparison with those of Fe-N_pl_-SAC (31.5 mV dec^−1^ for SRR, and 67.3 mV dec^−1^ for SER) and Fe-N_pc_-SAC (30.2 mV dec^−1^ for SRR and 71.1 mV dec^−1^ for SER), clearly demonstrating the kinetic advantage of Fe-N_pd_-SAC in the sulfur redox process.

The sulfur reduction kinetics were further investigated under scanning rates from 0.1 to 0.5 mV s^−1^ between 1.7 and 2.8 V (vs. Li/Li^+^). The contour plots of the CV patterns for Fe-N_pd_-SAC, Fe-N_pl_-SAC and Fe-N_pc_-SAC (Fig. S28) show the magnitude of peak intensities. It is evident that the cell with Fe-N_pd_-SAC electrode exhibits a higher current response than Fe-N_pl_-SAC and Fe-N_pc_-SAC electrodes. Meanwhile, the diffusion rate based on the CV curve was fitted, revealing that the cell with Fe-N_pd_-SAC exhibits the fastest ion transport kinetics, consistent with the analysis above, which mainly arises from the more favorable interaction of pyridine-N with Li ions than the other counterparts (Fig. S29). Even under conditions of limited structural differences, consistent trends in interfacial reaction behavior can still be observed, thereby demonstrating that the N-coordination environment exerts a systematic regulatory impact on LiPSs conversion.

### Electrochemical performances of Li-S batteries

Figure 4a exhibits the cycling performances of Li-S batteries based on three different catalysts. Fe-N_pd_-SAC electrode displays a higher capacity retention rate (95%) after 100 cycles as compared to Fe-N_pl_-SAC (86.2%) and Fe-N_pc_-SAC (82.3%) electrodes. SEM images of the cycled electrodes show the surface of Fe-N_pd_-SAC is fairly smooth without obvious cracking and agglomeration compared with that of S/Fe-N_pl_-SAC and S/Fe-N_pc_-SAC (Fig. S30), manifesting the restrained polysulfide shuttling and reduced reaction polarization. The electrochemical impedance spectroscopy (EIS) revealed that the Fe-N_pd_-SAC showcases the smallest electrode resistance (*R*_Ω_) and the charge transfer resistance (*R*_ct_) (Fig. S31), confirming its favorable interfacial kinetics.

**Figure 4.**
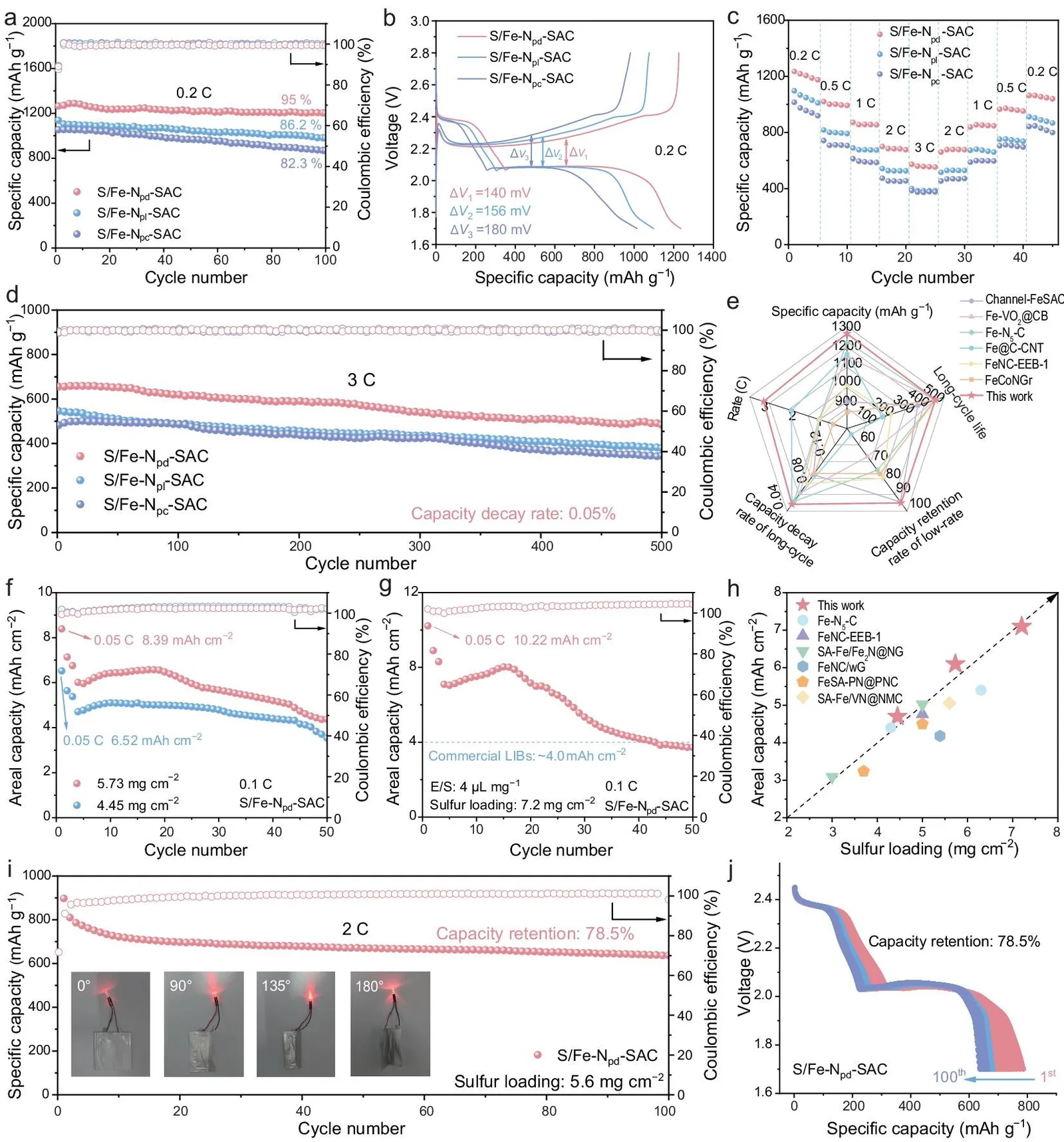
Electrochemical performances of Li-S batteries with S/Fe-N_4_-SAC electrodes. (a) Cycling performances of S/Fe-N_pd_-SAC, S/Fe-N_pl_-SAC and S/Fe-N_pc_-SAC electrodes at 0.2 C for 100 cycles. (b) GCD profiles at 0.2 C. (c) Rate performances of S/Fe-N_pd_-SAC, S/Fe-N_pl_-SAC and S/Fe-N_pc_-SAC electrodes. (d) Long cyclic performances of S/Fe-N_pd_-SAC, S/Fe-N_pl_-SAC and S/Fe-N_pc_-SAC electrodes at 3.0 C. (e) Comparison of electrochemical performances between this work (S/Fe-N_pd_-SAC) and previously reported studies with various Fe SACs. (f) Cycling performances of S/Fe-N_pd_-SAC electrode with high sulfur loadings. (g) Cycling performances of S/Fe-N_pd_-SAC electrode with a sulfur loading of 7.2 mg cm^−2^ and an E/S ratio of 4 μL mg^–1^. (h) Comparison of electrochemical performances with high sulfur loadings. (i) Cycling performances of Li-S pouch cell with S/Fe-N_pd_-SAC electrode. (j) GCD profiles of Li-S pouch cell.

The galvanostatic charge/discharge (GCD) curves of different electrodes show that the Fe-N_pd_-SAC electrode demonstrates a highest capacity (1265 mAh g^−1^) and the lowest voltage gap (140 mV) among three samples (Fig. 4b), verifying the excellent reaction kinetics of polysulfide conversion. The Fe-N_pd_-SAC electrode delivers superior capacities of 1235.3, 1020.3, 872.2, 699.9 and 572.7 mAh g^−1^ at 0.2, 0.5, 1.0, 2.0 and 3.0 C (Fig. 4c), respectively. When the current density returns to 0.2 C, a high capacity of 1063.6 mAh g^−1^ can be restored, demonstrating good electrochemical reversibility (Fig. S32). The Fe-N_pd_-SAC electrode has a stable discharge platform, indicating the best rate performance (Fig. S33). The cycling performance of the Li-S batteries at 1.0 C (Fig. S34) reveals that the Fe-N_pd_-SAC electrode delivers an initial capacity of 843.7 mAh g^−1^ and maintains a discharge capacity of 568.4 mAh g^−1^ after 500 cycles, showing a capacity decay rate of 0.067% per cycle. Even at a high rate of 3.0 C, Fe-N_pd_-SAC electrode still sustains a stable cycling operation with a low capacity decay rate of 0.05% per cycle after 500 cycles, outperforming Fe-N_pl_-SAC and Fe-N_pc_-SAC counterparts (Fig. 4d). The improvements in cycling stability are ascribed to the accelerated sulfur redox processes. Note that the electrochemical performances of Fe-N_pd_-SAC electrode compare favorably with most previous reported Li-S battery counterparts employing Fe single-atom catalyst mediators (Fig. 4e and Table S3) [26–31].

Optimizing the electrochemical performance under high sulfur loading is essential for the development of Li-S batteries for practical applications. In this regard, the batteries with the sulfur loading of 5.73 and 4.45 mg cm^−2^ showed initial areal capacities of 6.1 and 4.7 mAh cm^−2^ at 0.1 C (Fig. 4f and Fig. S35), along with an outstanding rate performance (Fig. S36). The low electrolyte/sulfur (E/S) ratio is another essential factor in achieving high-energy-density Li-S batteries [32,33]. To this end, the cell performance was evaluated under an E/S ratio of 4 µL mg^−1^ and a sulfur loading of 7.2 mg cm^−2^ at 0.1 C (Fig. 4g). The Fe-N_pd_-SAC electrode showed an excellent initial areal capacity of 7.1 mAh cm^−2^, transcending the typical areal capacity of commercial Li-ion batteries (4.0 mAh cm^−2^). The corresponding GCD curve at the first cycle (0.05 C) shows a stable discharge plateau, indicating excellent cycling ability of Fe-N_pd_-SAC electrode under the high sulfur loading (Fig. S37). Note that the phenomenon with respect to the continuous increased Coulombic efficiency even beyond 100% might stem from the interplay of more active material participating, progressive stabilization of electrochemical processes, and LiPSs shuttling in sulfur loading-elevated and electrolyte-depleted configurations. Overall, the performance metrics of Fe-N_pd_-SAC electrode compared favorably with previously reported Li-S batteries with Fe single-atom catalysis electrodes (Fig. 4h and Table S4) [28,30,34–38]. Finally, the practical potential of Fe-N_pd_-SAC was demonstrated by constructing a flexible Li-S pouch cell, which can continuously power a light emitting-diode at different bending angles (Fig. 4i inset). The pouch cell delivers a high capacity of 810.3 mAh g^−1^ at 2.0 C with the capacity retention of 78.5% after 100 cycles (Fig. 4i and j), suggesting the effectiveness of Fe-N_pd_-SAC in improving sulfur utilization under practical conditions.

### Investigating the catalytic mechanism of Fe-N_pd_-SAC

DFT calculation was carried out to investigate the influence of coordination environment on sulfur reaction kinetics. The molecular orbital hybridization between Fe *d*-orbital and N *p*-orbital was analyzed to reveal the influence of different coordination nitrogen species. Taking Fe-N_pd_-SAC and Fe-N_pl_-SAC as examples, the spin polarizations of Fe are in opposite directions to satisfy energy minimization according to the DOS and molecular orbital interaction (Fig. 5a). The interaction between Fe and N can be bifurcated into spin-down and spin-up. For Fe-N_pd_-SAC, the spin-down empty Fe *d*_*z*^2^_ orbital interacts with the occupied N–C *π* orbital and forms the unoccupied (*d*_*z*^2^_ − *π*)* and occupied *d*_*z*^2^_ orbital after Li adsorbed on Fe-N_pd_-SAC (Fig. 5b). However, the spin-up occupied *d*_*z*^2^_ orbital interacts with the empty π* orbital, forming the unoccupied (*d*_*z*^2^_ − π*)* and occupied *d*_*z*^2^_ orbital. The corresponding wave functions interaction was embedded in the projected density of states (PDOS). During this process, the d orbitals are non-degenerate orbitals with energy barrier between orbitals. In contrast, for Fe-N_pl_-SAC, *d*_*z*^2^_ and *d*_*x*^2^−*y*^2^_, *d_xz_* and *d_yz_* orbitals are degenerate orbitals after Li adsorption (Fig. 5c and d). Moreover, the spin-up occupied *d_yz_* orbital interacts with the empty π* orbital, forming the unoccupied (*d_yz_* − π*)* and occupied *d_yz_* orbital. Based on the above analysis, we found that the lower the degeneration of *d*-orbital, the larger *d*-orbital participates in the charge transfer which provides enhanced catalytic activity.

**Figure 5.**
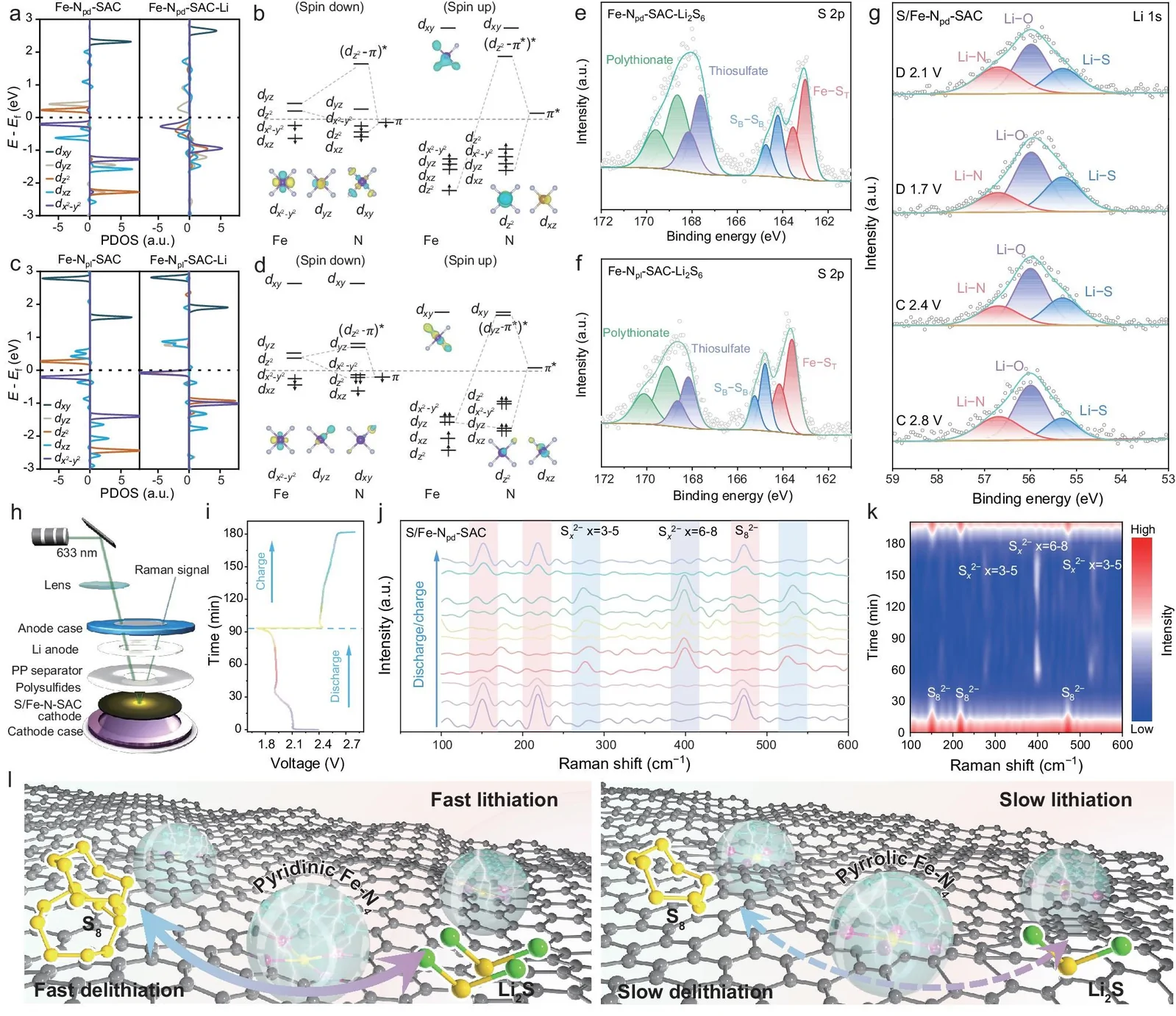
Investigation of catalytic mechanisms for active Fe-N_4_-SAC. (a) PDOS profile of Fe-N_pd_-SAC. (b) Spin-down and spin-up *d*-orbital interactions with corresponding wave functions for Fe-N_pd_-SAC. (c) PDOS profile of Fe-N_pl_-SAC. (d) *d*-orbital interactions for Fe-N_pl_-SAC. XPS S 2*p* spectra of (e) Fe-N_pd_-SAC and (f) Fe-N_pl_-SAC with Li_2_S_6_ dosage after 3 h. (g) XPS Li 1*s* spectra of S/Fe-N_pd_-SAC electrode during the cycling. (h) Schematic illustration of Li-S cell configuration constructed for *operando* Raman measurement. (i) The GCD curve at the first full cycle of 0.2 C for *operando* Raman measurement. *Operando* Raman spectra of S/Fe-N_pd_-SAC electrode on the (j) separator interface and (k) electrode interface. (l) Schematic diagram showing the catalysis mechanism of Fe-N_pd_-SAC in Li-S chemistry.

The efficient adsorption and rapid conversion of LiPSs play a vital role in Li-S chemistry. In this sense, the polysulfide adsorption behaviors were examined. Our studies indicated that the Li_2_S_6_ solution turns colorless after 3 h upon the addition of Fe-N_pd_-SAC (Fig. S38), while retaining a slight yellow color for Fe-N_pl_-SAC and Fe-N_pc_-SAC, visually demonstrating more favorable capture of polysulfides by Fe-N_pd_-SAC.

The XPS analysis of specimen after adsorption further confirms the interaction between Fe-N_4_-SAC and LiPSs. The S 2*p* spectra upon the adsorption test could be deconvoluted into four peaks at 163/163.6, 164.2/165.8, 167.6/168.2, and 168.7/169.6 eV (Fig. 5e), which are ascribed to terminal S in LiPSs (S_T_^−1^), bridged S in LiPSs (S_B_^0^), thiosulfate and polythionate species, respectively. The existence of S-metal bonds indicates that the LiPSs are chemically conjugated on the Fe sites of Fe-N_pd_-SAC [39]. The thiosulfate is generated by the reaction of Fe-N_pd_-SAC with LiPSs, while the polythionate could be derived from the conversion between thiosulfate and LiPSs [2,40]. The coexistence of these sulfurated species confirms that Fe-N_pd_-SAC possesses a strong affinity toward LiPSs due to its stronger *d-p* orbital hybridization. Upon interaction with Fe-N_pl_-SAC and Fe-N_pc_-SAC, the S 2*p* spectra of LiPSs showed significantly lower S_T_^−1^ ratios than those of Fe-N_pd_-SAC (Fig. 5f and Fig. S39). These results reveal that Lewis acid-base interaction exists between Fe-N_pd_-SAC and LiPSs, accordingly enhancing the capture and anchor of LiPSs.

The Li 1*s* spectra of Fe-N_4_-SAC with Li_2_S_6_ show the peak at 55.5 and 56.8 eV assigned to the Li–S and Li–N bonding, suggesting the strong chemical anchoring between active N atoms and the terminal Li atoms in LiPSs (Fig. S40). Clearly, Fe-N_pd_-SAC exhibits the largest Li–N/Li–S ratio value (Fig. S41), suggesting that the pyridine-N therein has the strongest interaction with the terminal Li in LiPSs. These results reveal that Lewis acid-base interaction exists between Fe-N_4_-SAC and LiPSs, accordingly enhancing the capture and anchor of LiPSs. The XPS analysis is consistent with theoretical calculations that the first shell layer of pyridine-N not only has a better affinity for Li, but its coordinated Fe sites achieve stronger adsorption of LiPSs through greater participation in the reactive *d*-orbitals.

*Ex situ* and *in situ* tests were used to further analyze the role of the Fe-N_pd_-SAC catalyst in the Li-S battery cycling process. Figure 5g shows the spectra of *ex situ* XPS of Li 1*s* during a full cycle of Fe-N_pd_-SAC electrode to decouple the role of N atoms in catalytic reactions. Three peaks corresponding to Li–S species (55.3 eV), Li–O species (56.0 eV) and Li–N species (56.7 eV) were observed. During the discharge process (D 2.1 V and 1.7 V), a decrease in the proportion of Li–N bond and an increase of Li-S bond are accompanied by the conversion of LiPSs to the discharge product Li_2_S, suggesting that the N atom plays a critical role in facilitating adsorption of LiPS and mediating Li^+^ transfer in Li-S batteries.

We employed *operando* Raman spectroscopy and *in situ* XRD to provide insight into the catalytic effect of Fe-N_pd_-SAC during the charging and discharging of Li-S batteries (Fig. 5h). The Raman signal was collected from the separator or Fe-N_pd_-SAC electrode interface, which can accurately provide feedback of the LiPSs deposited. Here, we employed Raman parameters based on a laser wavelength of 633 nm and a laser intensity of 1% for measurement during a single charge/discharge cycle. Figure 5i shows the GCD curve of *operando* Raman spectra at the first full cycling of 0.2 C. On the separator interface (Fig. 5j), three Raman characteristic peaks of S_8_^2−^ (located at ∼148, 213, and 474 cm^−1^) emerge at the beginning of the discharge process (first plateau at ∼2.1 V). As the voltage decreases, the long-chain LiPSs (S*_x_*^2−^, *x* = 6–8, ∼400 cm^−1^) and short-chain LiPSs (S*_x_*^2−^, *x* = 3–5, ∼279 cm^−1^) signals appear successively and eventually disappear. The Raman signals show a reversible trend with increasing voltage during charging, indicating an excellent sulfur redox conversion process in the battery. For the Fe-N_pd_-SAC electrode interface (Fig. 5k), Raman shift intensities of S_8_^2−^, S_6_^2−^, and S_3_^2−^ peaks gradually decline in the discharge process and regenerate during charging, revealing the LiPSs trapping and catalyzing ability of Fe-N_pd_-SAC electrocatalysts. The comparison between electrode-side and separator-side sulfur species provides direct evidence that the Fe–N–C catalyst not only accelerates reaction kinetics but also enhances interfacial reaction selectivity to promote localized conversion processes.

*In situ* XRD clearly shows *α*-S_8_ crystal peak at ∼23.2° (JCPDS 008–0247) at the beginning of the discharge process, which gradually weakens with the emergence of Li_2_S characteristic peaks at ∼27° (JCPDS 023–0369) (Fig. S42), illustrating the reversible solid-liquid-solid phase conversion process from *α*-S_8_ to Li_2_S in S/Fe-N_pd_-SAC electrode. These *in situ* characterizations further demonstrate the efficient catalytic ability of Fe–N–C catalyst. Figure 5l summarizes the origin of the Li-S catalysis activity of the Fe-N_pd_-SAC. For the formation of pyridine-N coordination, the degeneration of *d*-orbital and the larger *d*-orbital participates in the charge transfer and thereby activates the Fe single atom sites, enhancing the catalytic activity for sulfur redox in Li-S chemistry.

## CONCLUSION

In summary, guided by DFT calculations, we precisely tailor the first-shell coordination nitrogen Fe–N_4_ SACs with pyridinic-N and pyrrolic-N enhanced sulfur redox kinetics. The Fe-N_pd_-SAC exhibits a *d*-band center closer to the Fermi energy level, with N_pd_ featuring a lower Li adsorption free energy and stronger Li–N_pd_  *s–p* orbital hybridization compared to N_pl_, which not only promotes LiPSs adsorption and conversion, but also facilitates faster Li-ion transport. Electrochemical tests confirm that Fe-N_pd_-SAC exhibit superior sulfur redox kinetics and enhanced ion transport, attributed to the largest *d*-orbital participation in the charge transfer. As a result, Fe-N_pd_-SAC electrode delivers outstanding electrochemical performance, achieving a high specific capacity of 1265 mAh g^−1^ and excellent stability with a capacity fading of < 0.05% per cycle. Furthermore, Li-S batteries incorporating this electrode demonstrate excellent areal capacity (7.1 mAh cm^−2^) and cyclic stability under an elevated sulfur loading (7.2 mg cm^−2^). This study identifies the pyridinic-type FeN_4_ as the more active Fe–N coordination motif promoting the sulfur redox kinetics and offers fundamental insights into the catalytic mechanism of M–N–C catalysts in Li-S batteries.

## METHODS

### Electrochemical tests of sulfur electrodes

The electrochemical performances of the standard CR2032 coin cells were tested using S/Fe-N_pd_-SAC, S/Fe-N_pl_-SAC and S/Fe-N_pc_-SAC as cathodes, Li foil as anode and Celgard 2400 membrane as the separator. The electrolyte contained 0.5 M LiTFSI in a solvent mixture of DOL and DME (1 : 1 in volume) with 0.5 M LiNO_3_. CV measurements at varied scan rates were performed on the CHI660D electrochemical workstation. GCD tests and rate/cycling performance were conducted on a LAND battery testing system with a voltage range of 1.7–2.8 V (vs. Li/Li^+^). The GITT experiment was carried out to confirm a purely kinetic effect. The batteries undergo a current pulse protocol at 0.1 C for 10 min with rested 20 min. The internal resistance can be quantified by the following relation equation: ∆*R*_internal_ (Ω) = |∆*V*_QOCV−CCV_|/*I*_applied_ where Δ*V*_QOCV−CV_ is the voltage difference between the points of quasi open-circuit voltage (QOCV) and closed-circuit voltage (CCV), and *I*_applied_ is the applied current.

### Characterizations

The crystal structures of products were characterized by XRD in Bruker D8 Advance Diffractometer with Cu-K*α* radiation. Morphologies and EDS mapping images were collected using Hitachi SU8010 SEM and Titan Themis Cubed G2300 TEM. The aberration-corrected STEM-HAADF images were taken by a TEM (FEI Titan3 G2 60–300) equipped with double spherical aberration correctors. XPS measurements were conducted on Escalab 250Xi Spectrophotometer using a monochromatic Al Kα X-ray source. Nitrogen adsorption/desorption isotherms were gained at 77 K on the Micromeritics Tristar II 3020 Accelerated Surface Area and Porosimetry System. Transition metal loading was analyzed by inductively coupled plasma optical emission spectrometry using an Optima 8000, Perkin Elmer.

### Theoretical calculations

DFT calculations were conducted using the Vienna ab initio Simulation Package [41] with the Perdew-Burke-Ernzerhof functional [42]. DFT-D2 correction was employed to describe the van der Waals force. Six models with different numbers of pyridine and pyrrole nitrogen were obtained by the restructuring of the graphene. The thickness of slab vacuum was set as 15 Å to avoid the interaction between periodic layers. The geometries and free energies were fully optimized with plane-wave cutoff energy of 500 eV, energy convergence of 10^–4^ eV and force convergence of 0.01 eV Å^–1^. The K-grid of Brillouin zone was 3 × 3 × 1 with Monkhorst–Pack algorithm. The binding energy was calculated by the Gibbs free energy difference between reactants and products with the consideration of the entropy and the zero-point energy correction. The charge density difference and Bader charge methods were employed for the analysis of the electron transfer. The CINEB calculations were performed to unveil the kinetic barrier of Li-ion diffusion [43].

## Supplementary Material

nwag433_Supplemental_File
