# Supplementary material for: Tuning primary shell nitrogen in Fe–N–C for enhanced sulfur redox electrocatalysis
Source: Natl Sci Rev. 2026 Jul 13;13(20):nwag433. doi: 10.1093/nsr/nwag433 (PMC13750157; doi:10.1093/nsr/nwag433)
Supplement: Supplementary file 1 [file nwag433_supplemental_file.pdf]

Supporting Information

Tuning primary shell nitrogen in Fe–N–C for enhanced sulfur redox electrocatalysis

METHOD

Preparation of Fe-N_{pd}-SAC

0.75 g of 2-methylimidazole ($C_4H_6N_2$) was mixed into the 15 mL deionized (DI) water with ultrasonic treatment for 10 min to form homogeneous solution. Then 0.08 g of Ferric nitrate nonahydrate ($Fe(NO_3)_3 \cdot 9H_2O$) and 0.15 g of carbon black (ECP600JD) were added to the above solution under ultrasonication. After stirring for 10 h at room temperature, the above-mixed solution underwent vacuum filtration treatment to obtain the black mixture powder. Subsequently, the black mixture powder was polymerized at 800 °C for 2 h with a heating rate of 3 °C min^{-1} under the Ar atmosphere. The obtained power was added to the 1 M HCl solution at 60 °C, which was stirred for 24 h to remove the Fe nanoparticle. Next, the obtained black powder was collected by centrifugation and washed three times with DI water. Finally, the Fe-N_{pd}-SAC was obtained by 24 h vacuum drying.

Preparation of Fe-N_{pl}-SAC

0.75 g of 4-dimethylaminopyridine ($C_7H_{10}N_2$) was mixed into the 15 mL DI water with ultrasonic treatment for 10 min to form homogeneous solution. Then 0.03 g of $Fe(NO_3)_3 \cdot 9H_2O$ and 0.15 g of ECP600JD were successively added into the clear $C_4H_6N_2$ solution under ultrasonication. After stirring for 10 h at room temperature, the above-mixed solution underwent vacuum filtration treatment to obtain the black mixture powder. Then, the black mixture powder was calcined at 800 ºC for 2 h under Ar atmosphere with the rising temperature rate of 3 ºC min^{-1}. After acid etching treatment with 1M HCl solution at 60 °C for 24 h, the Fe-N_{pl}-SAC was obtained.

Preparation of Fe-N_{pc}-SAC

0.7 g of dicyandiamide ($C_2H_4N_4$) was mixed into the 15 mL DMF with ultrasonic treatment for 10 min to form homogeneous solution. Then 0.04 g of iron phthalocyanine ($C_{32}H_{16}FeN_8$) and 0.15 g of ECP600JD were successively added into the clear $C_2H_4N_4$ solution under ultrasonication. After stirring for 10 h at room temperature, the above-mixed solution underwent vacuum filtration treatment to obtain the black mixture powder. Then, the black mixture powder was calcined at 800 ºC for 2 h under Ar atmosphere with the rising temperature rate of 3 ºC min^{-1}. After acid etching treatment with 1M HCl solution at 60 °C for 24 h, the Fe-N_{pc}-SAC was obtained.

Preparation of S/Fe-N_{pd}-SAC, S/Fe-N_{pl}-SAC and S/Fe-N_{pc}-SAC

Sulfur composites were prepared by the melt-diffusion method. Sulfur and host materials (Fe-N_{pd}-SAC, Fe-N_{pl}-SAC and Fe-N_{pc}-SAC) were mixed at a ratio of 4:1, which were ground in a mortar for 30 min. Then the mixture was put into a glass bottle and heated at 155 °C for 12 h to enable the diffusion of the sulfur substance into the pores of the host materials.

Li_2S_6 symmetric cell assembly and measurements

Li_2S_6 symmetrical cells were assembled in a glovebox filled with Ar atmosphere. 0.2 mol L^{-1} Li_2S_6 solution was prepared by mixing Li_2S and sulfur with a molar ratio of 1:5 in the DME/1,3-dioxolane (DOL) solution containing 0.5 mol L^{-1} LiTFSI and 0.2 mol L^{-1} $LiNO_3$. The Fe-N_{pd}-SAC/CP, Fe-N_{pl}-SAC/CP and Fe-N_{pc}-SAC/CP were prepared by dissolving 10 mg Fe-N_{pd}-SAC, Fe-N_{pl}-SAC and Fe-N_{pc}-SAC in 5 mL ethanol and dropping them evenly on the carbon paper (CP), which enabled a mass loading of 0.5 mg cm^{-2}. The two identical electrodes were assembled into a standard CR2032 coin cell with a Celgard 2400 membrane served as the separator. 40 μL as-prepared Li_2S_6 solution was used as the electrolyte. CV tests of symmetric cells were performed at a scan rate of 0.5 and 50 mV s^{-1} with the voltage ranging from –1 to 1 V.

Li_2S nucleation tests

0.2 mol L^{-1} Li_2S_8 solution was prepared by dissolving Li_2S and sulfur at a molar ratio of 1:7 in tetraglyme solution, followed by a vigorous stirring at 60 °C for 24 h. As-prepared Fe-N_{pd}-SAC/CP, Fe-N_{pl}-SAC/CP and Fe-N_{pc}-SAC/CP were employed as cathode, lithium foil as anode and Celgard 2400 membrane as the separator. 25 μL Li_2S_8 (0.2 mol L^{-1}) electrolyte was added into the cathode and 25 μL LiTFSI (0.5 mol L^{-1}) was added into the anode side. The assembled cells were galvanostatically discharged to 2.06 V at 0.112 mA and kept potentiostatically at 2.05 V to obtain the current response as a function of time until the current decreased to below 10^{-5} A.

PITT measurement

The coin cell for the PITT test was assembled using Fe-N_{pd}-SAC/CP, Fe-N_{pl}-SAC/CP and Fe-N_{pc}-SAC/CP as the working electrode and Li foil as the anode. 40 μL of catholyte (0.5 mol L^{-1} Li_2S_8 dissolved in DOL/DME with 0.5 mol L^{-1} LiTFSI and 0.5 mol L^{-1} $LiNO_3$) and 5 μL of blank electrolyte were dropped onto the cathode and anode side, respectively. The PITT test was conducted on the PARSTAT 3000A electrochemical workstation. The cell was potentiostatically discharged from the open voltage to 1.9 V and then charged to 2.5 V with 50.0 mV per interval.

Li_2S_6 visualized adsorption tests

Li_2S_6 solution with a concentration of 3.5 mmol L^{-1} was prepared by mixing Li_2S and sulfur in the 1,2-dimethoxyethane (DME) solution at a molar ratio of 1:5, followed by the continuous stirring until completely dissolved. Then, Fe-N_{pd}-SAC, Fe-N_{pl}-SAC and Fe-N_{pc}-SAC (5 mg) were added to 2 mL as-prepared Li_2S_6 solutions. The whole process was carried out in a glove box.

Supporting Figure

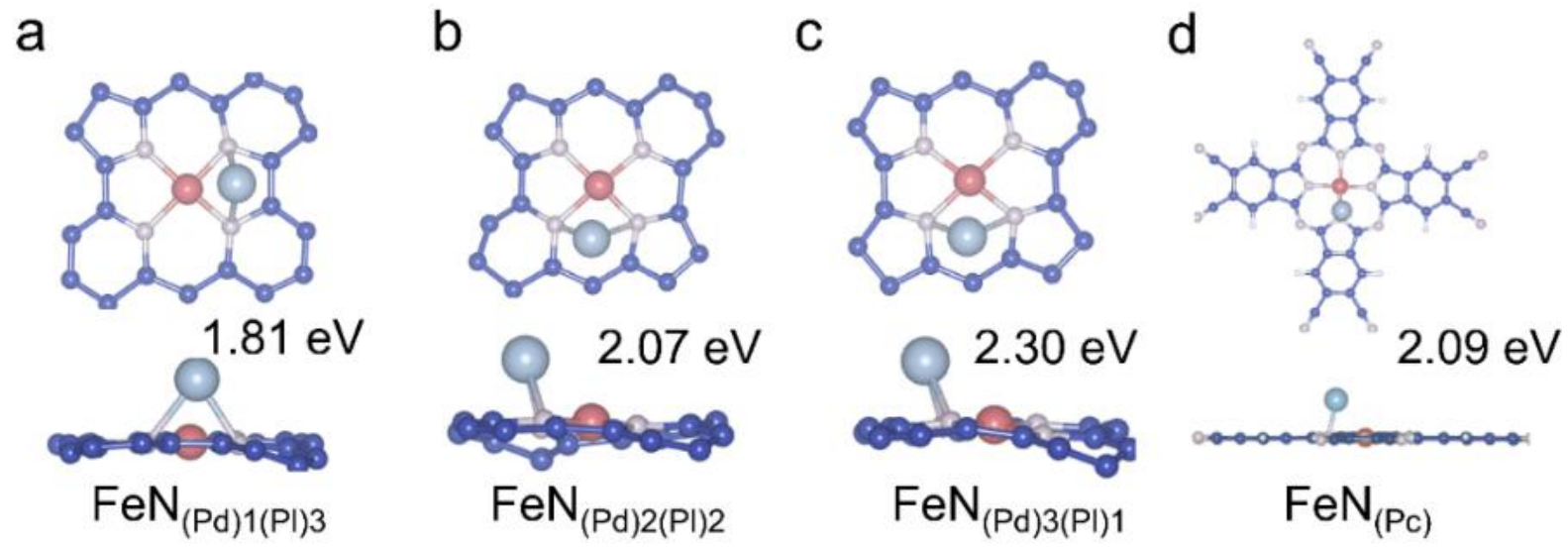

Figure S1. Structural models and adsorption energy values of Li atom for (a) $FeN_{(Pd)1(Pl)3}$, (b) $FeN_{(Pd)2(Pl)2}$, (c) $FeN_{(Pd)3(Pl)1}$, and (d) $FeN_{(Pc)}$.

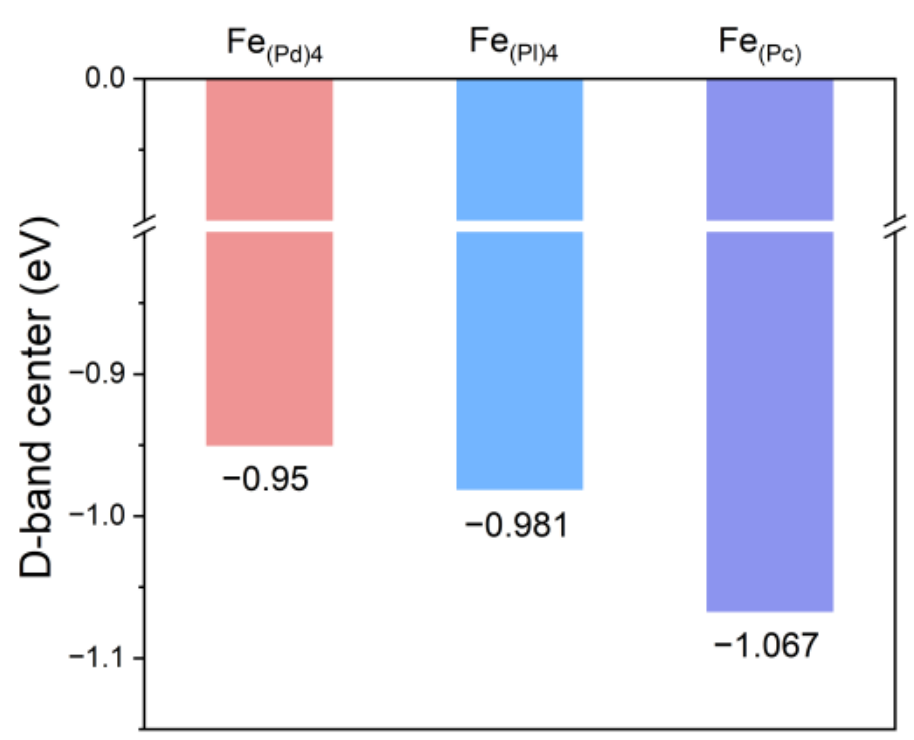

Figure S2. *d*-band center information of $FeN_{(Pd)4}$, $FeN_{(Pl)4}$ and $FeN_{(Pc)}$.

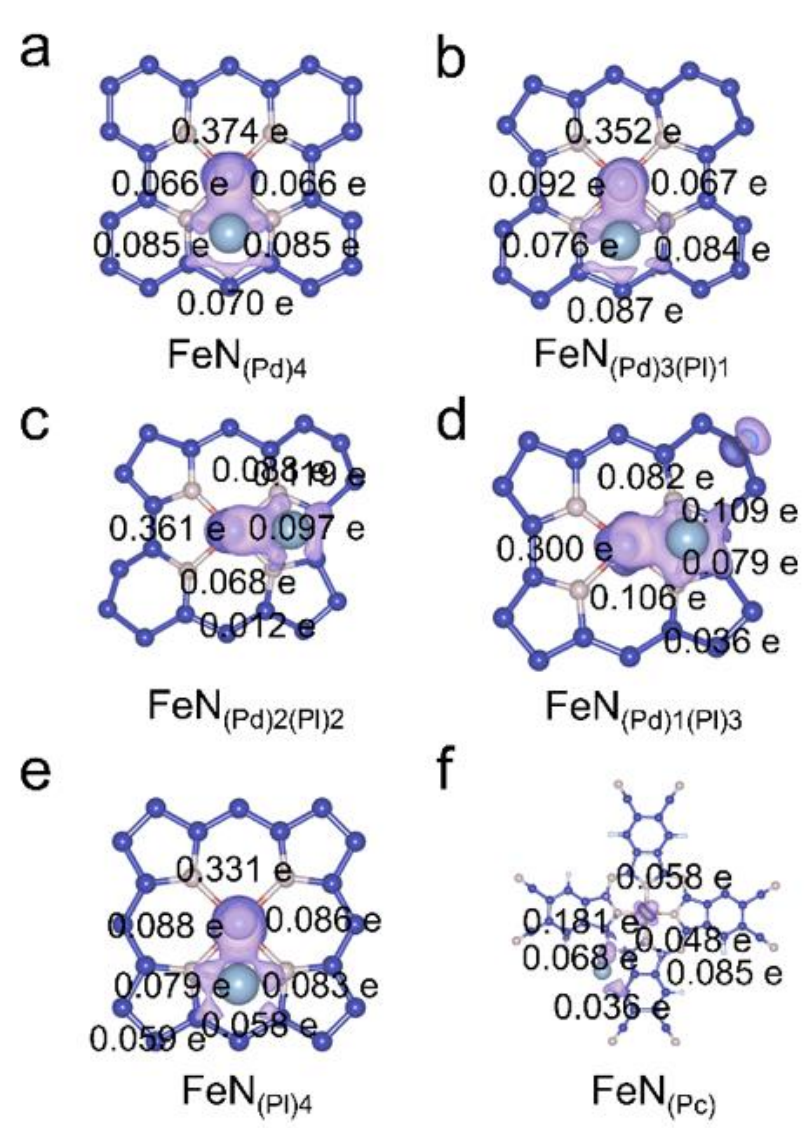

Figure S3. Charge density difference calculations for (a) $FeN_{(Pd)4}$, (b) $FeN_{(Pd)3(Pl)1}$, (c) $FeN_{(Pd)2(Pl)2}$, (d) $FeN_{(Pd)1(Pl)3}$, (e) $FeN_{(Pl)4}$, and (f) $FeN_{(Pc)}$.

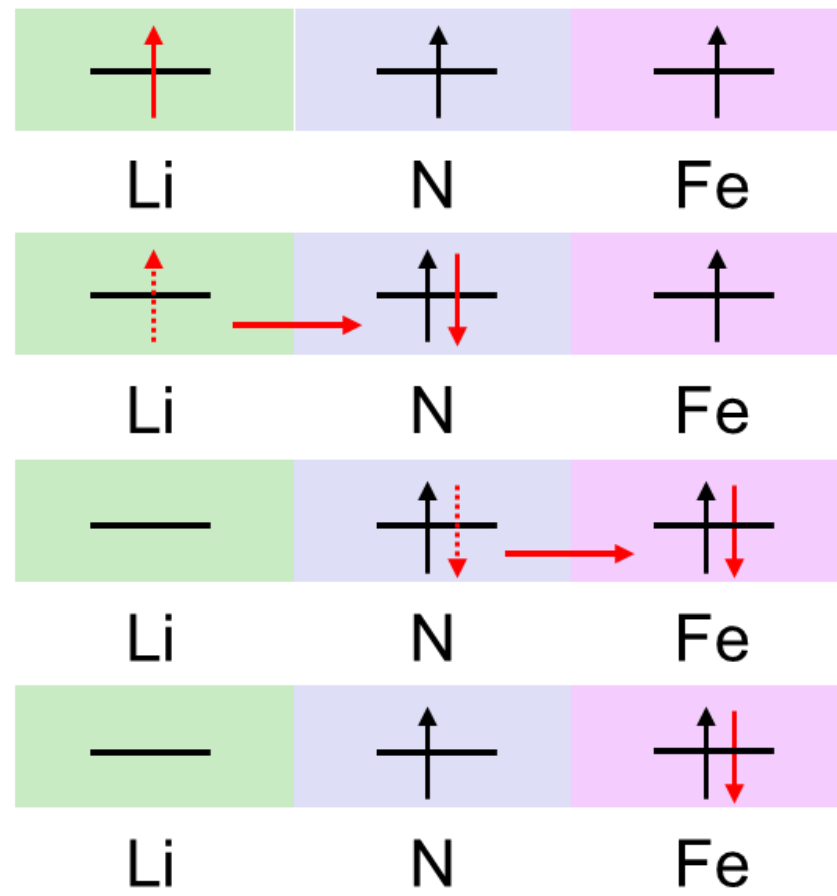

Figure S4. The electron transfer demonstration between Li atom and Fe−N−C moiety.

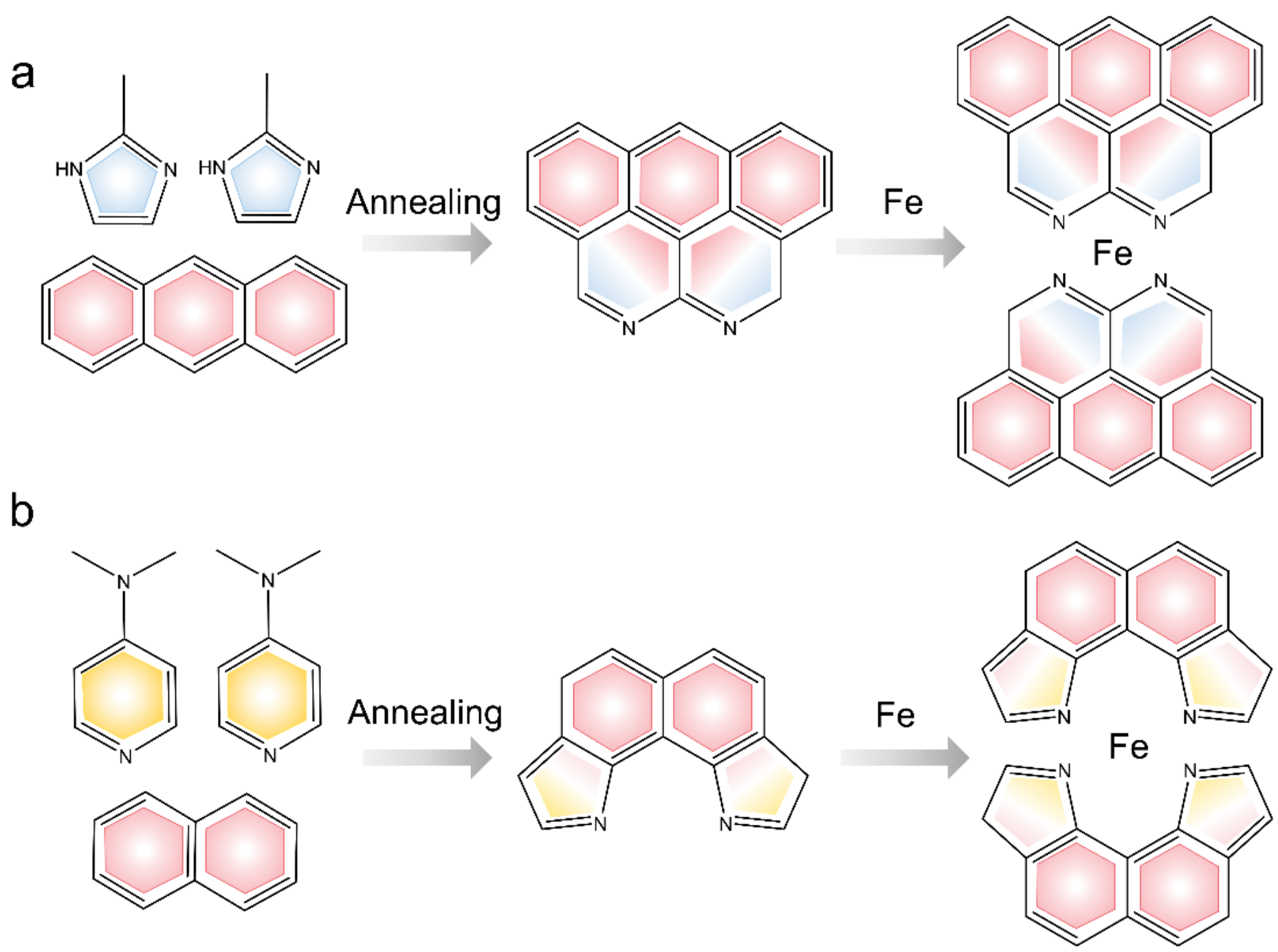

Figure S5. The proposed formation process of (a) pyridine-type FeN_4 and (b) pyrrole-type FeN_4.

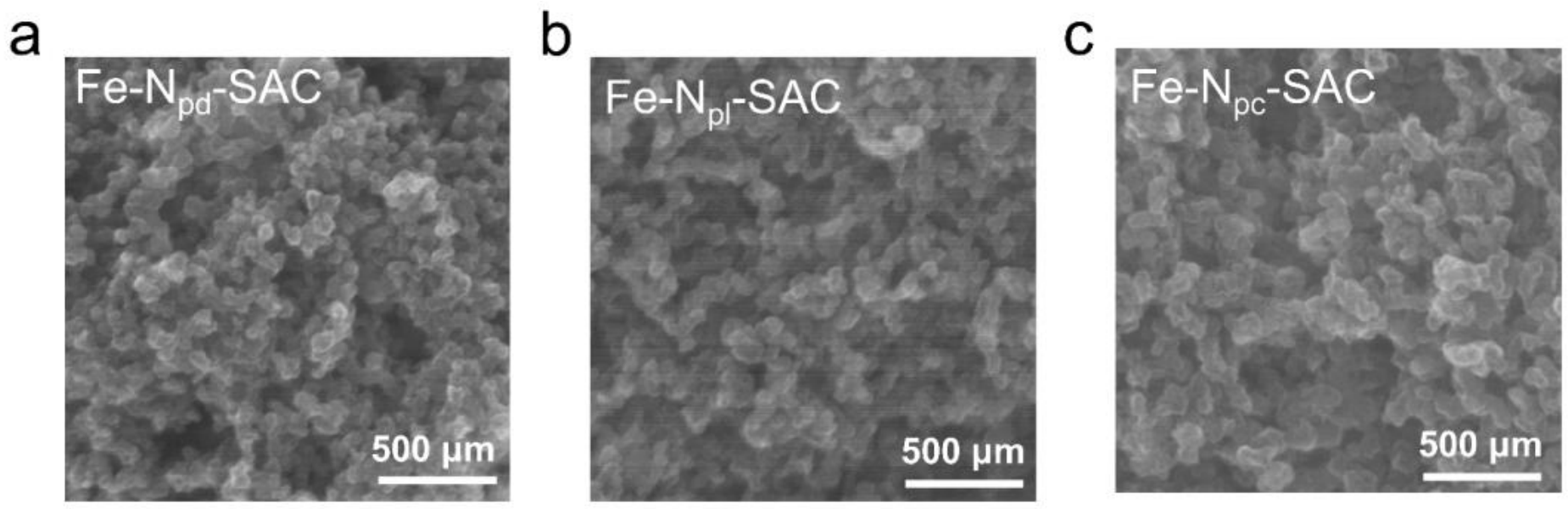

Figure S6. SEM images of (a) Fe-N_{pd}-SAC, (b) Fe-N_{pl}-SAC and (c) Fe-N_{pc}-SAC.

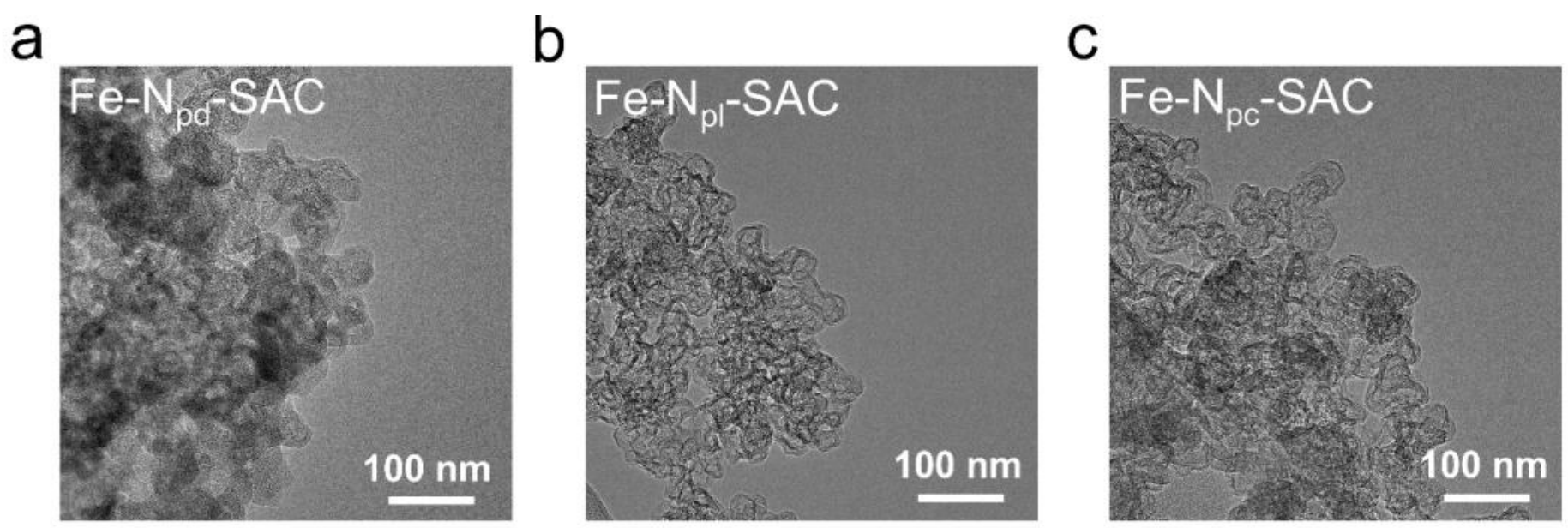

Figure S7. Low-magnification TEM images of (a) Fe-N_{pd}-SAC, (b) Fe-N_{pl}-SAC and (c) Fe-N_{pc}-SAC.

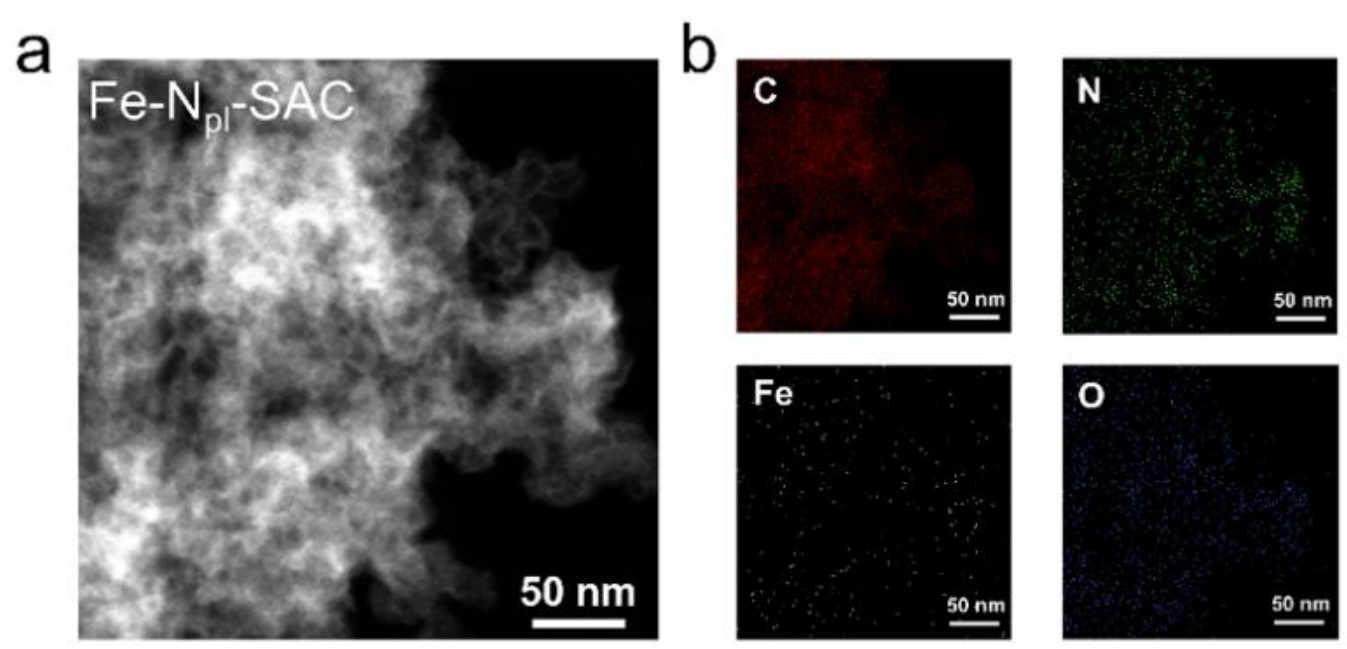

Figure S8. (a) Representative STEM image and (b) corresponding elemental maps of Fe-N_{pl}-SAC.

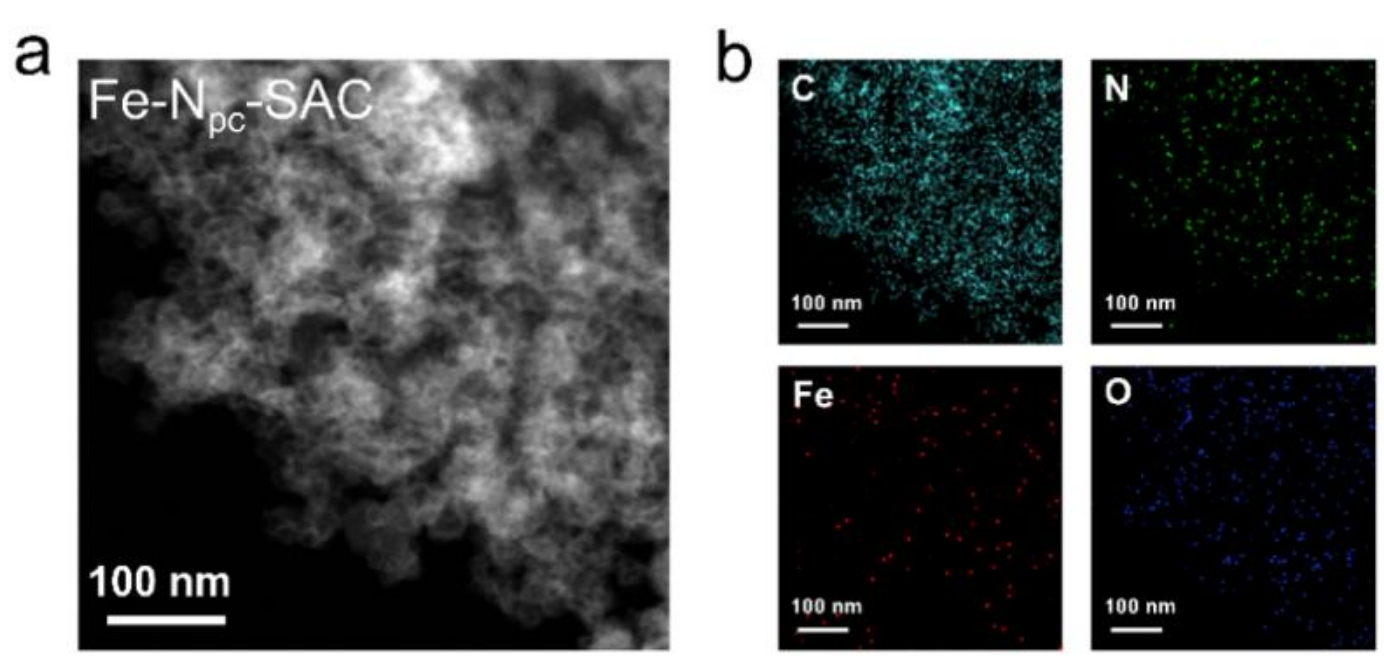

Figure S9. (a) Representative STEM image and (b) corresponding elemental maps of Fe-N_{pc}-SAC.

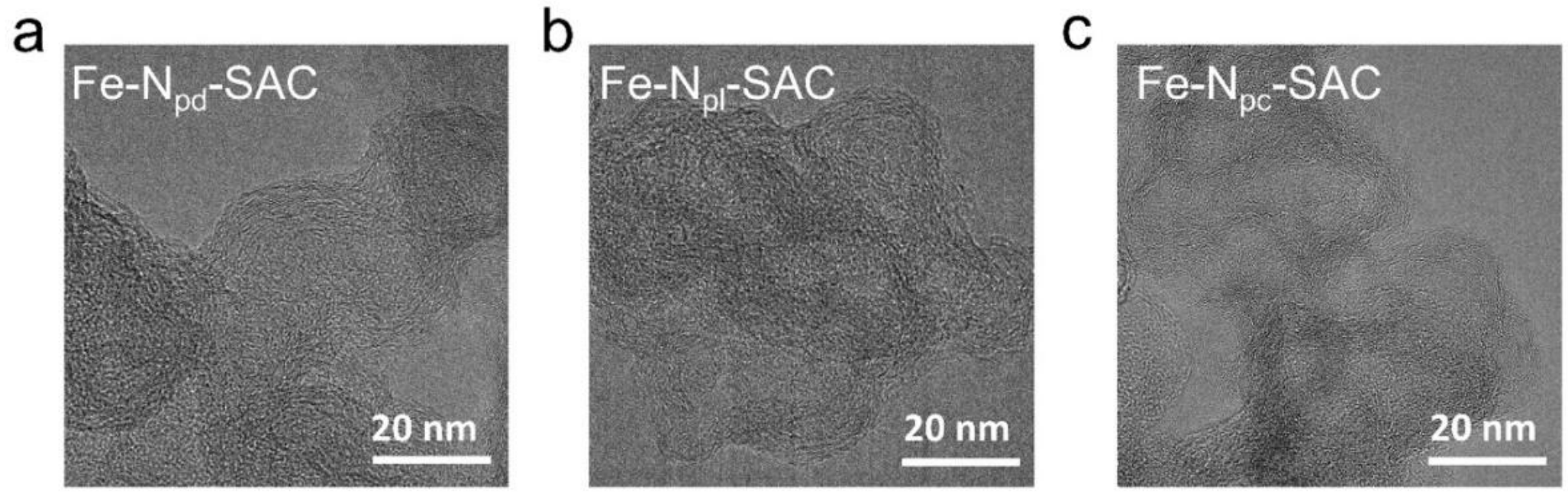

Figure S10. HRTEM images of (a) Fe-N$_{pd}$-SAC, (b) Fe-N$_{pl}$-SAC and (c) Fe-N$_{pc}$-SAC.

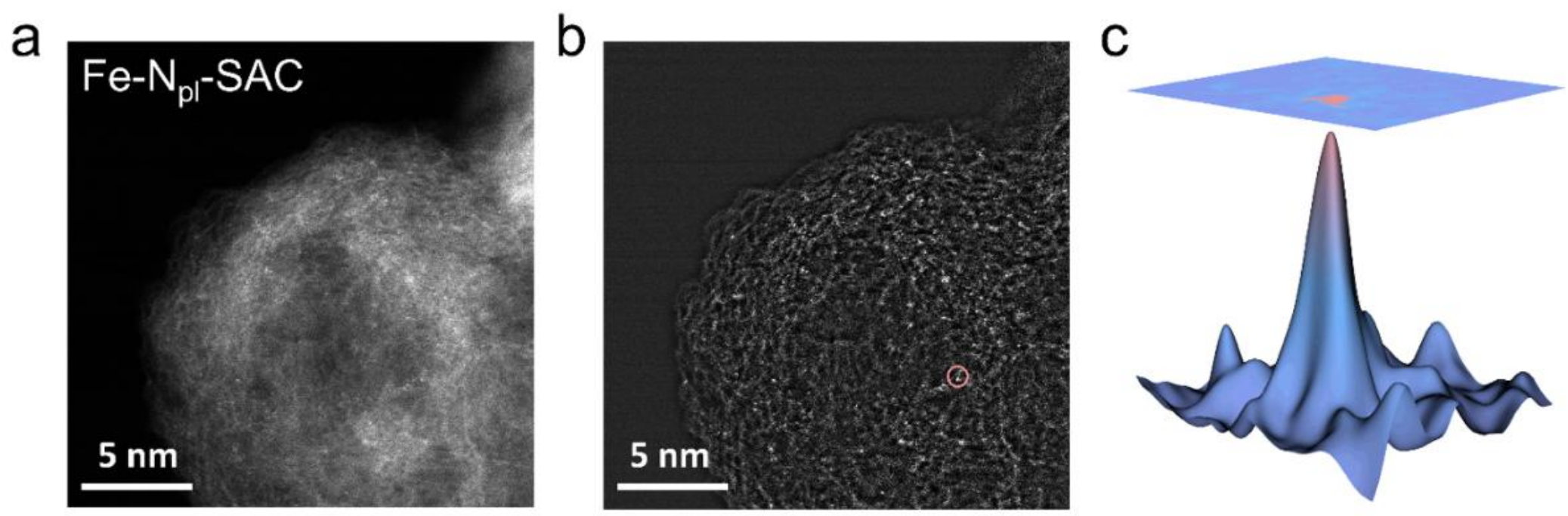

Figure S11. HAADF-STEM images of Fe-N$_{pl}$-SAC.

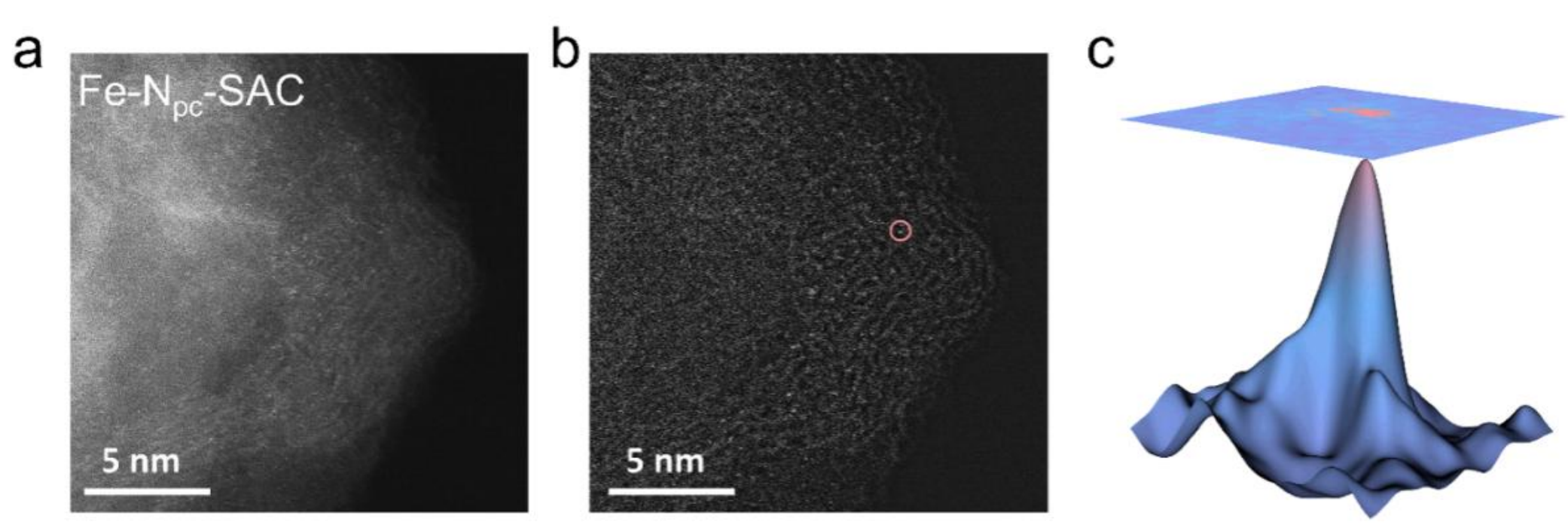

Figure S12. HAADF-STEM images of Fe-N$_{pc}$-SAC.

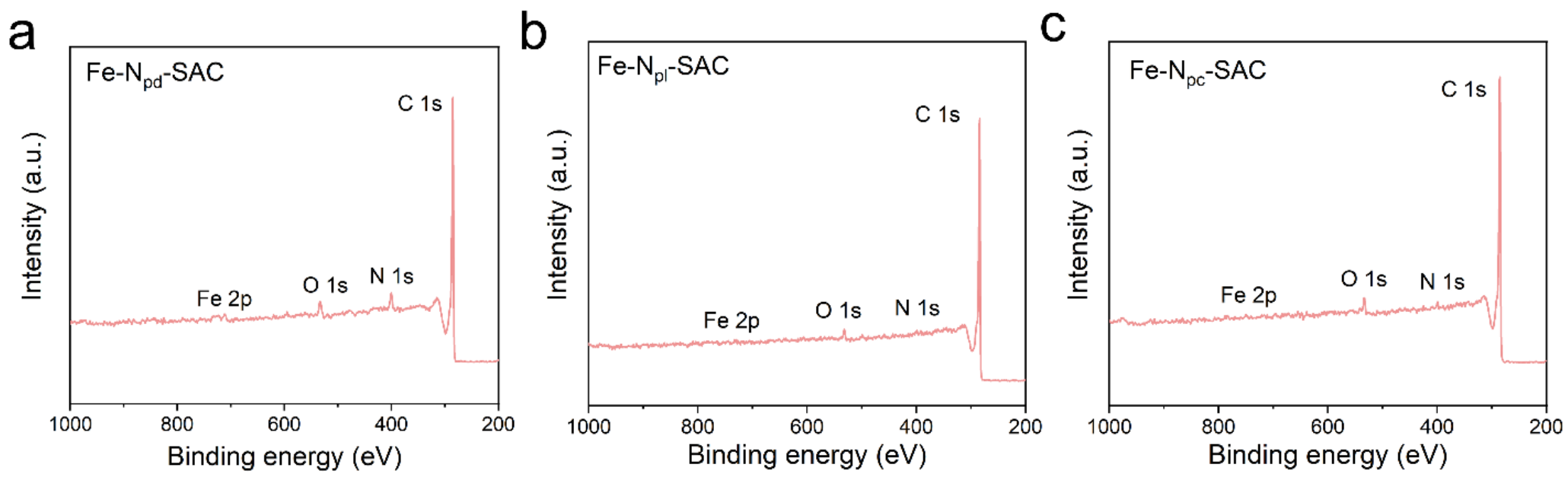

Figure S13. XPS survey spectra of (a) Fe-N$_{pd}$-SAC, (b) Fe-N$_{pl}$-SAC and (c) Fe-N$_{pc}$-SAC.

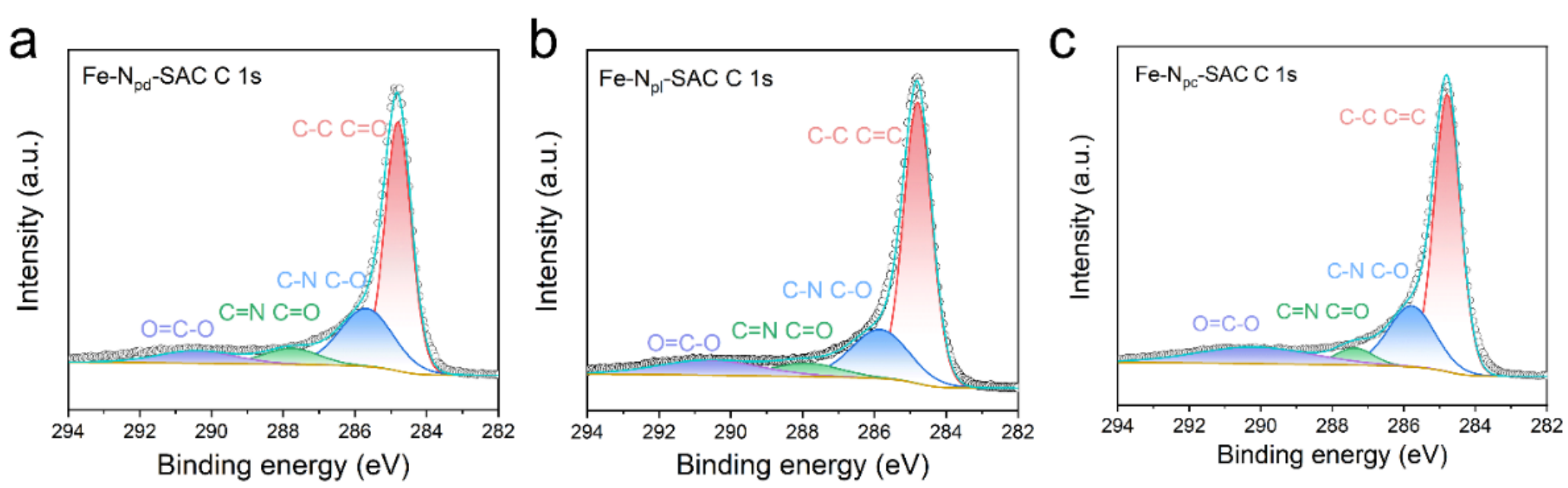

Figure S14. XPS C 1*s* spectra of (a) Fe-N$_{pd}$-SAC, (b) Fe-N$_{pl}$-SAC and (c) Fe-N$_{pc}$-SAC.

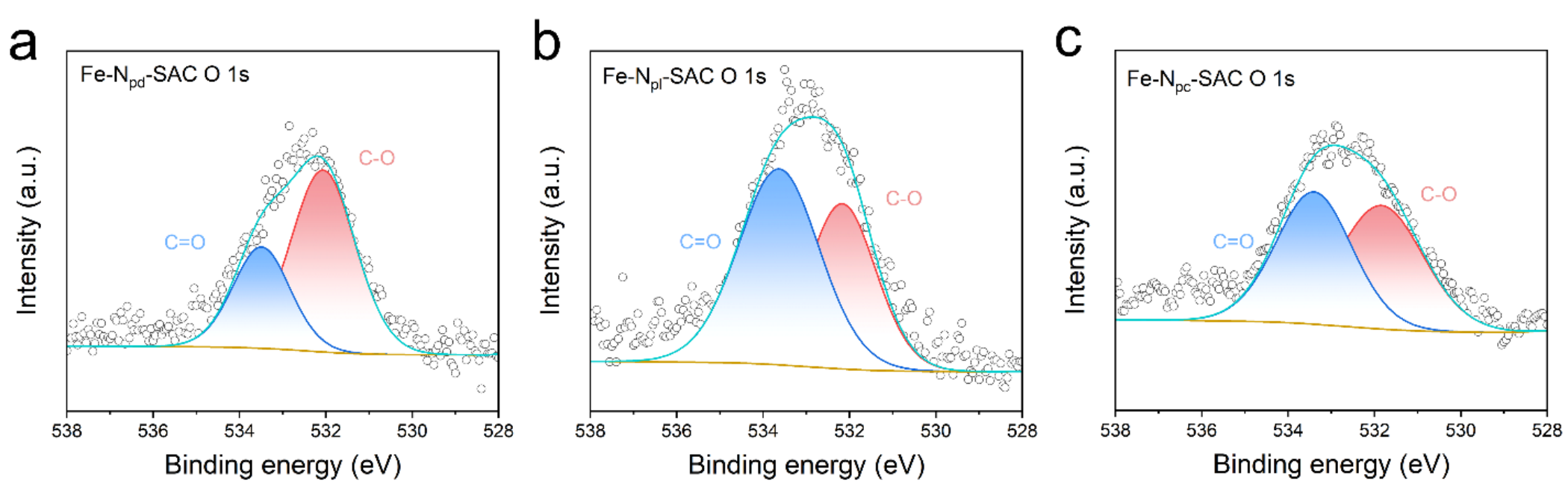

Figure S15. XPS O 1*s* spectra of (a) Fe-N$_{pd}$-SAC, (b) Fe-N$_{pl}$-SAC and (c) Fe-N$_{pc}$-SAC.

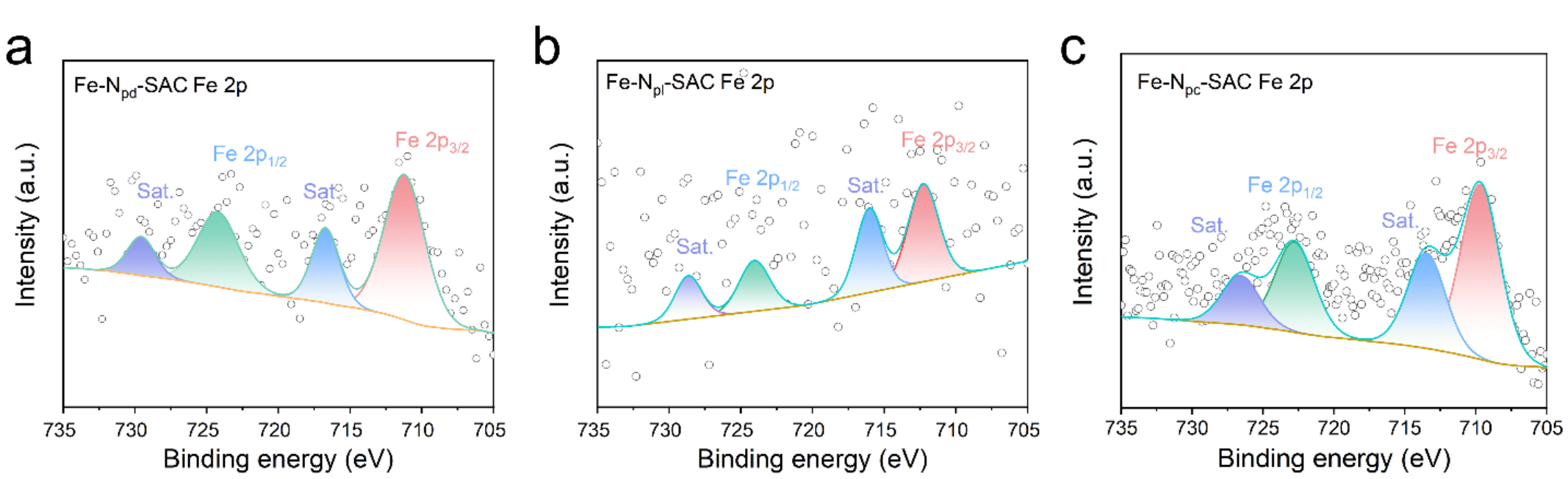

Figure S16. XPS Fe 2*p* spectra of (a) Fe-N$_{pd}$-SAC, (b) Fe-N$_{pl}$-SAC and (c) Fe-N$_{pc}$-SAC.

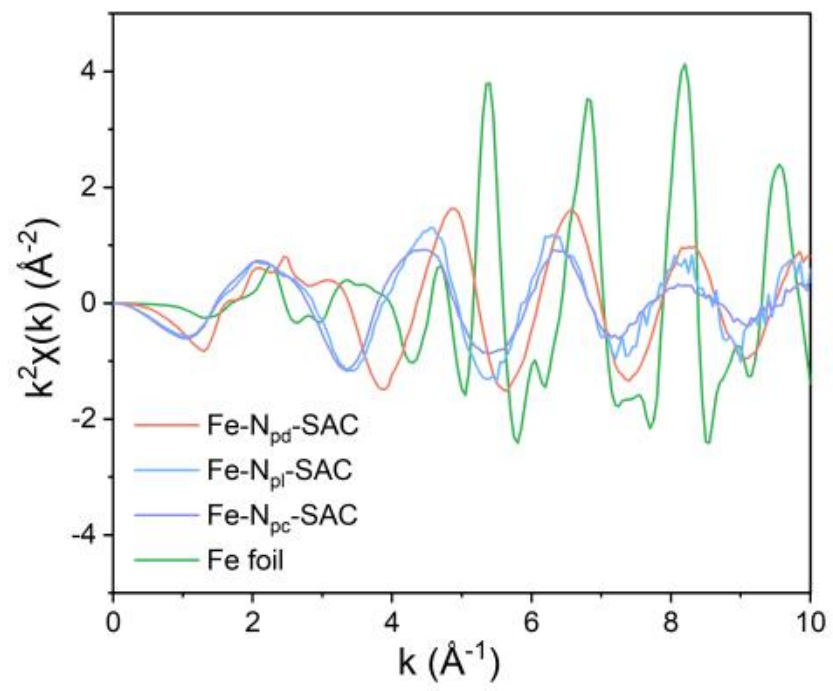

Figure S17. The k^2-weighted EXAFS spectra of Fe-N_{pd}-SAC, Fe-N_{pl}-SAC , Fe-N_{pc}-SAC and Fe foil.

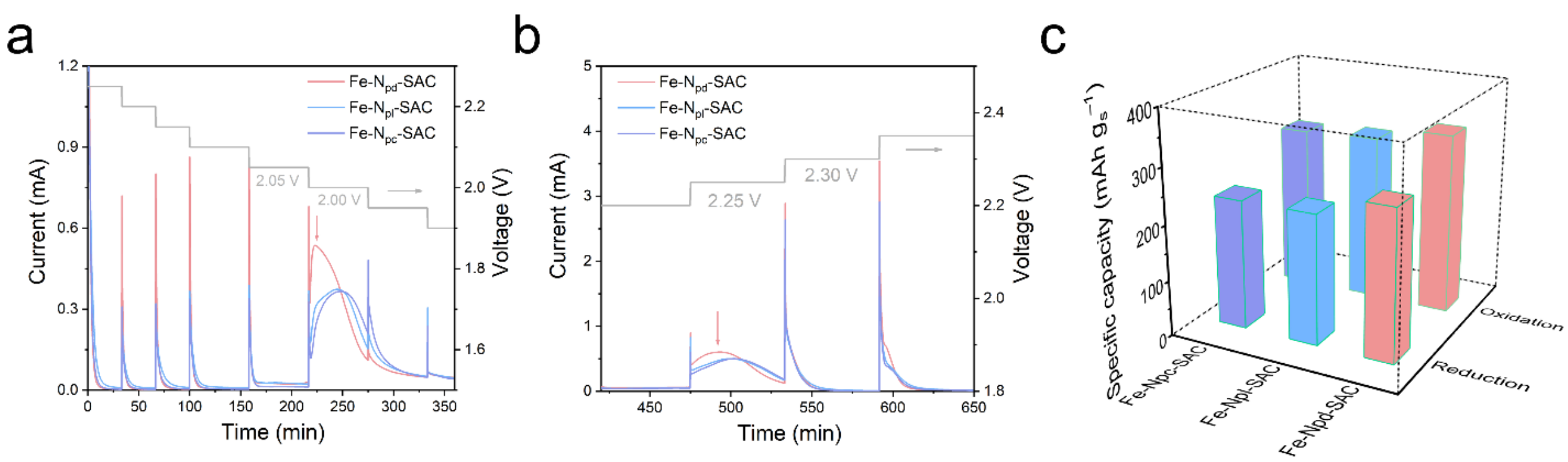

Figure S18. Current response in the PITT test at the constant potential of Fe-N_{pd}-SAC, Fe-N_{pl}-SAC and Fe-N_{pc}-SAC in (a) discharge and (b) charge process. (c) The calculated capacity of Li_2S nucleation and Li_2S dissolution under PITT operation of Fe-N_{pd}-SAC, Fe-N_{pl}-SAC and Fe-N_{pc}-SAC.

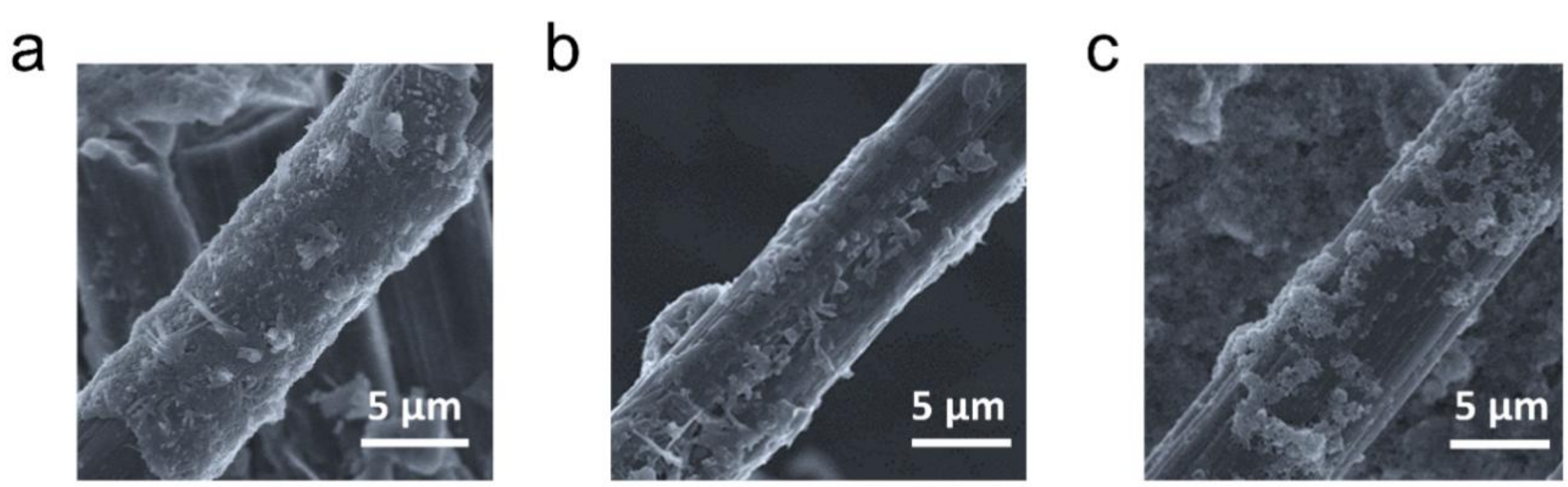

Figure S19. SEM images of the dissected cells after potentiostatically discharging at 2.05 V of (a) Fe-N_{pd}-SAC, (b) Fe-N_{pl}-SAC and (c) Fe-N_{pc}-SAC.

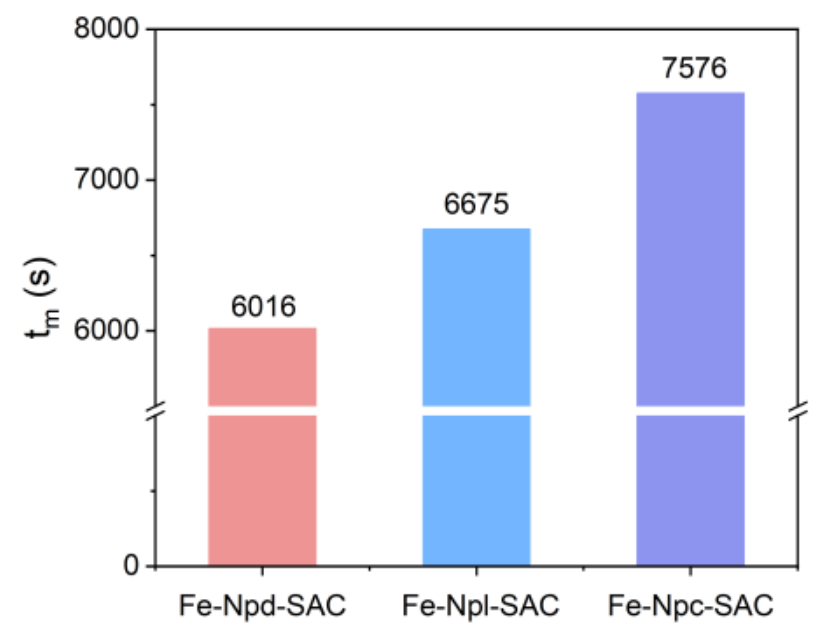

Figure S20. The Li_2S nucleation time (t_m) of Fe-N_{pd}-SAC, Fe-N_{pl}-SAC and Fe-N_{pc}-SAC.

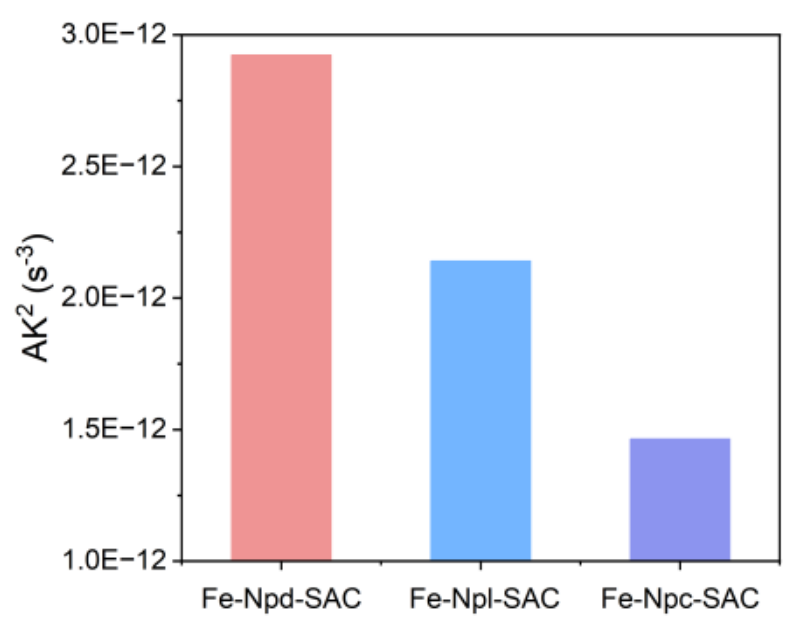

Figure S21. The Ak^2 value of Fe-N_{pd}-SAC, Fe-N_{pl}-SAC and Fe-N_{pc}-SAC.

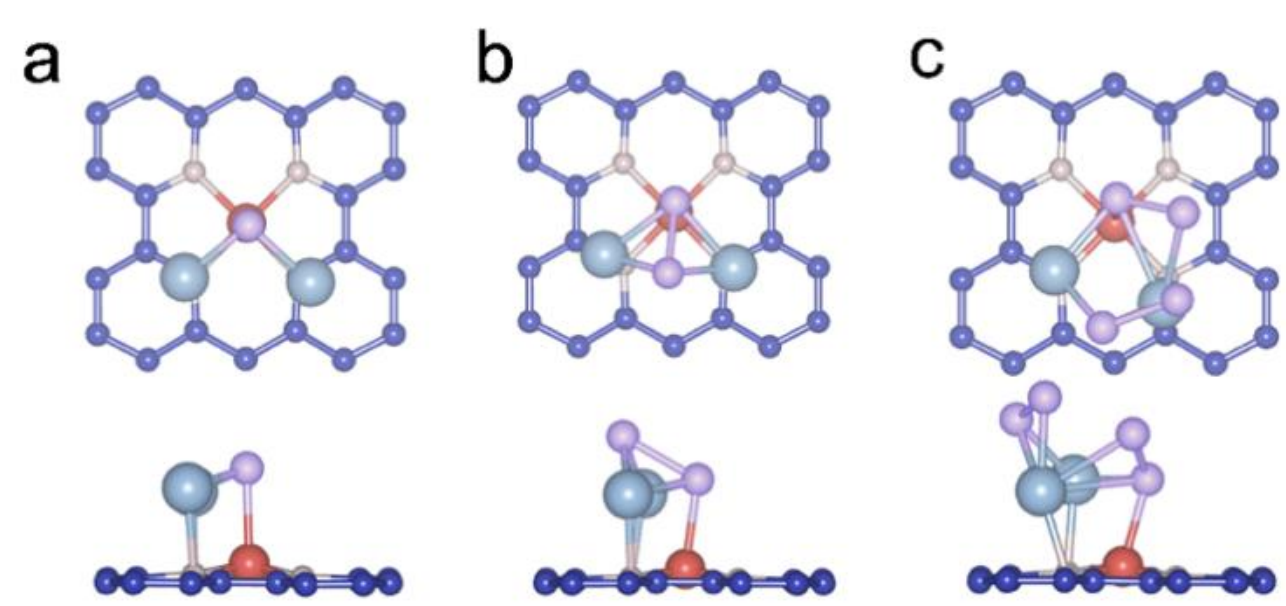

Figure S22. Optimized structures of Fe-N_{pd}-SAC for SRR with (a) Li_2S, (b) Li_2S_2 and (c) Li_2S_4.

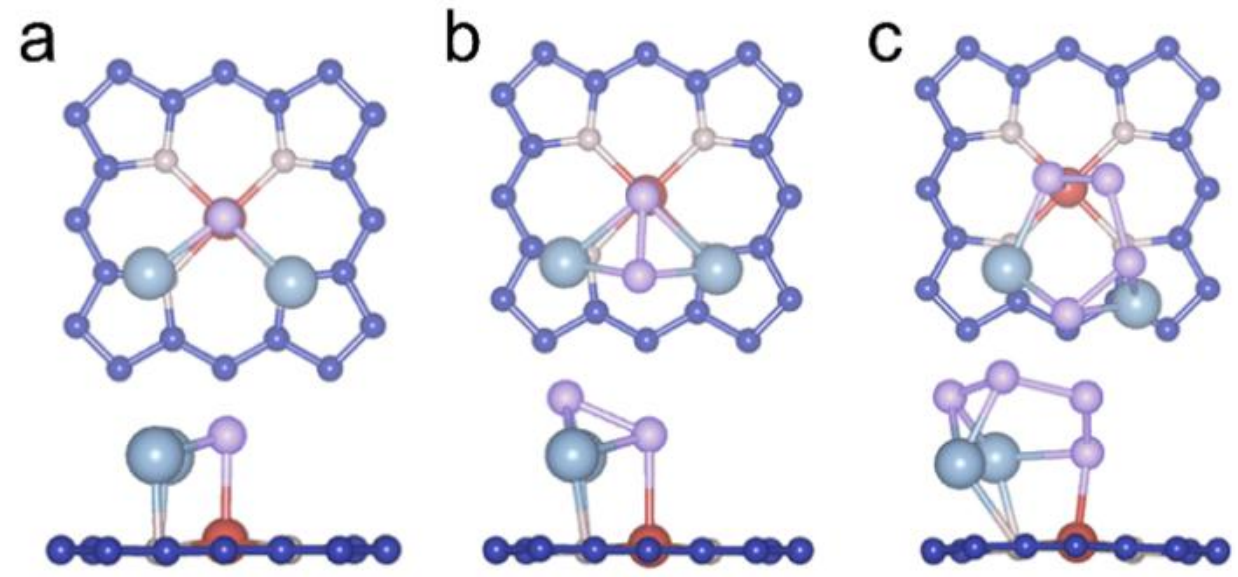

Figure S23. Optimized structures of Fe-N_{pl}-SAC for SRR with (a) Li_2S, (b) Li_2S_2 and (c) Li_2S_4.

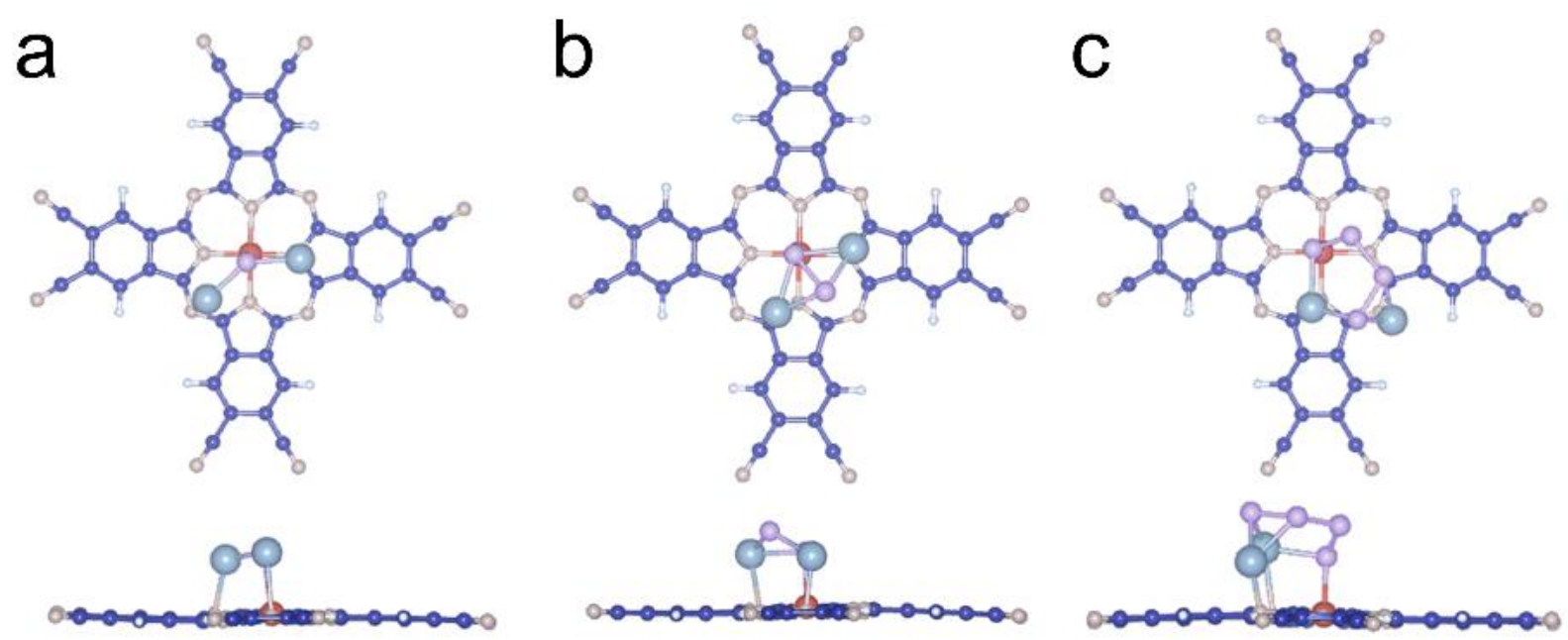

Figure S24. Optimized structures of Fe-N_{pc}-SAC for SRR with (a) Li_2S, (b) Li_2S_2 and (c) Li_2S_4.

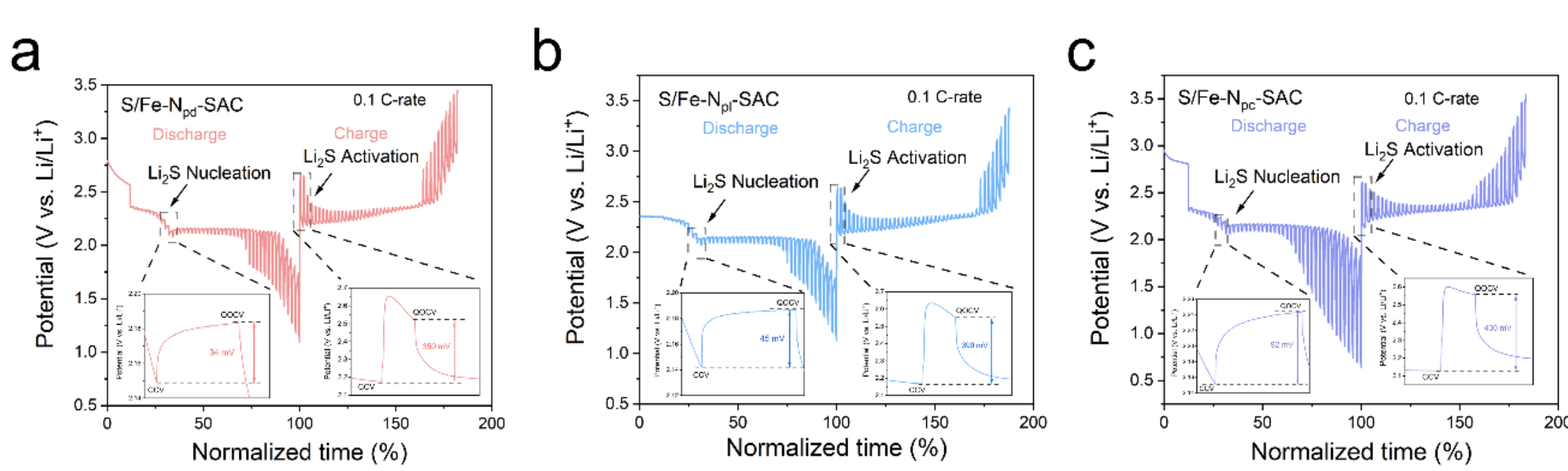

Figure S25. GITT voltage profiles of (a) S/Fe-N_{pd}-SAC, (b) S/Fe-N_{pl}-SAC and (c) S/Fe-N_{pc}-SAC electrodes at 0.1 C.

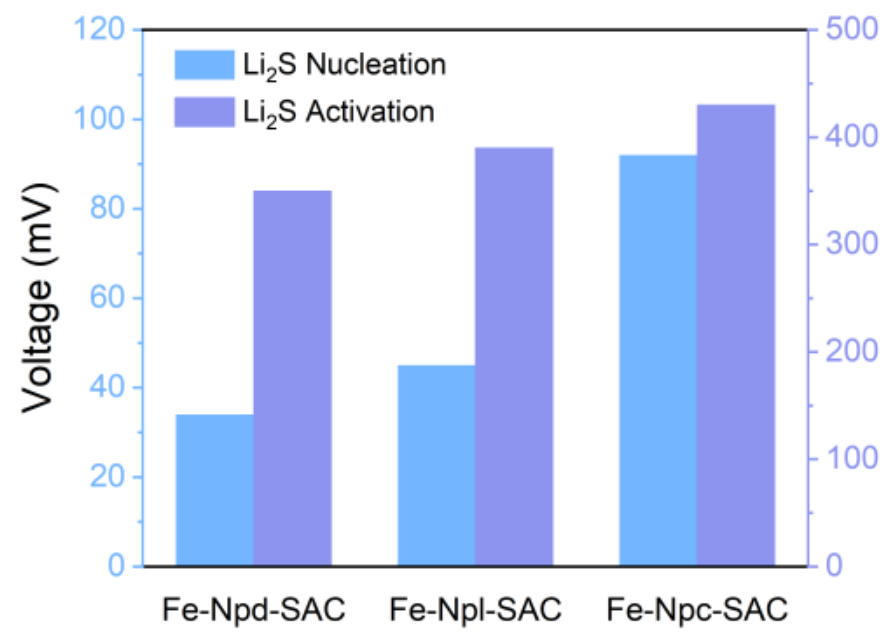

Figure S26. The potential difference between quasi open-circuit voltage and closed-circuit voltage at Li_2S nucleation and Li_2S activation point.

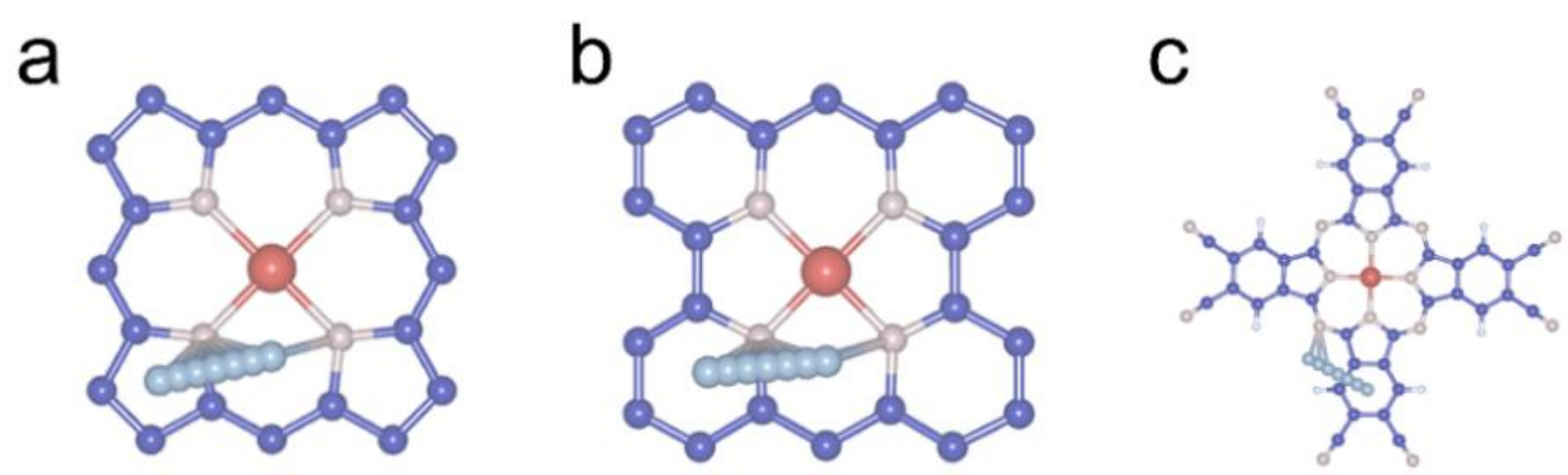

Figure 27. Li diffusion pathway for (a) Fe-N_{pd}-SAC, (b) Fe-N_{pl}-SAC and (c) Fe-N_{pc}-SAC.

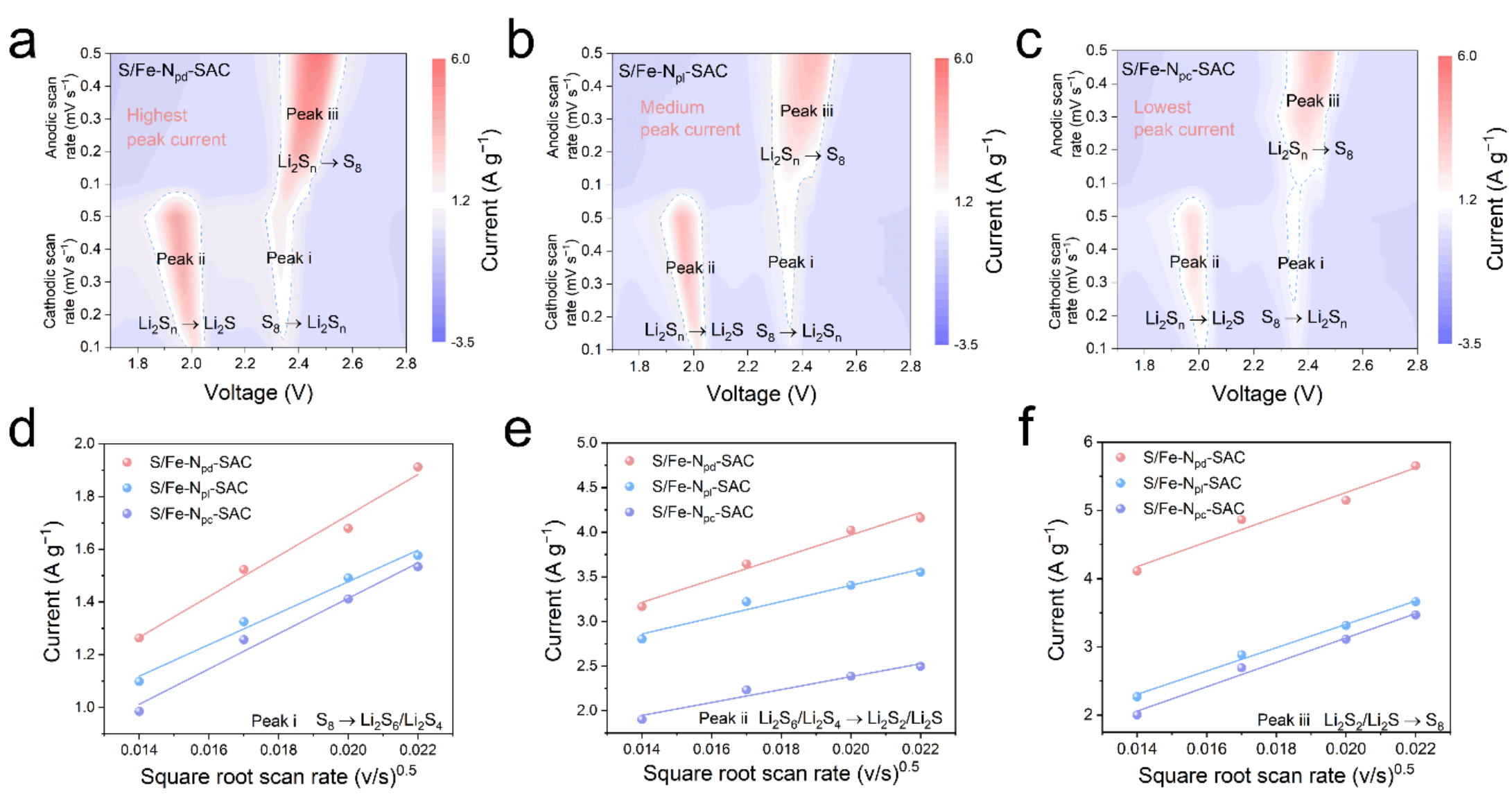

Figure S28. Contour plots of CV patterns at different scan rates with (a) S/Fe-N_{pd}-SAC, (b) S/Fe-N_{pl}-SAC and (c) S/Fe-N_{pc}-SAC electrodes. (d-f) Li-ion diffusion properties probed by the plots of CV peak currents *vs.* square root of the scan rates.

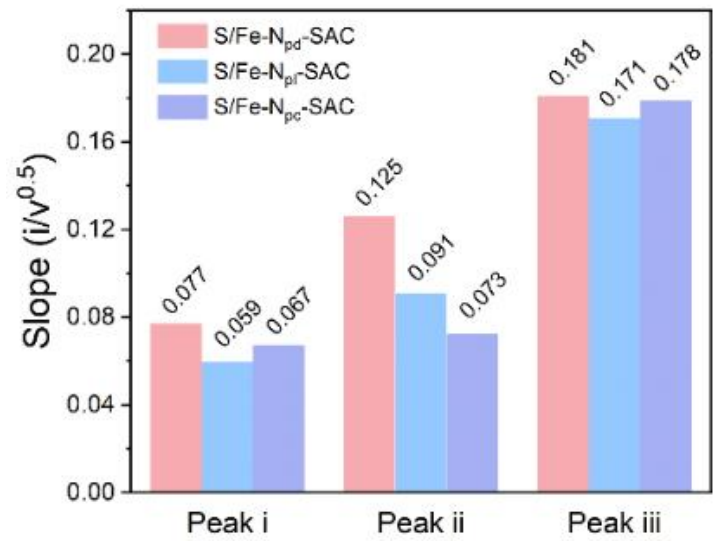

Figure S29. Li-ion diffusion slopes of three peaks from CV patterns.

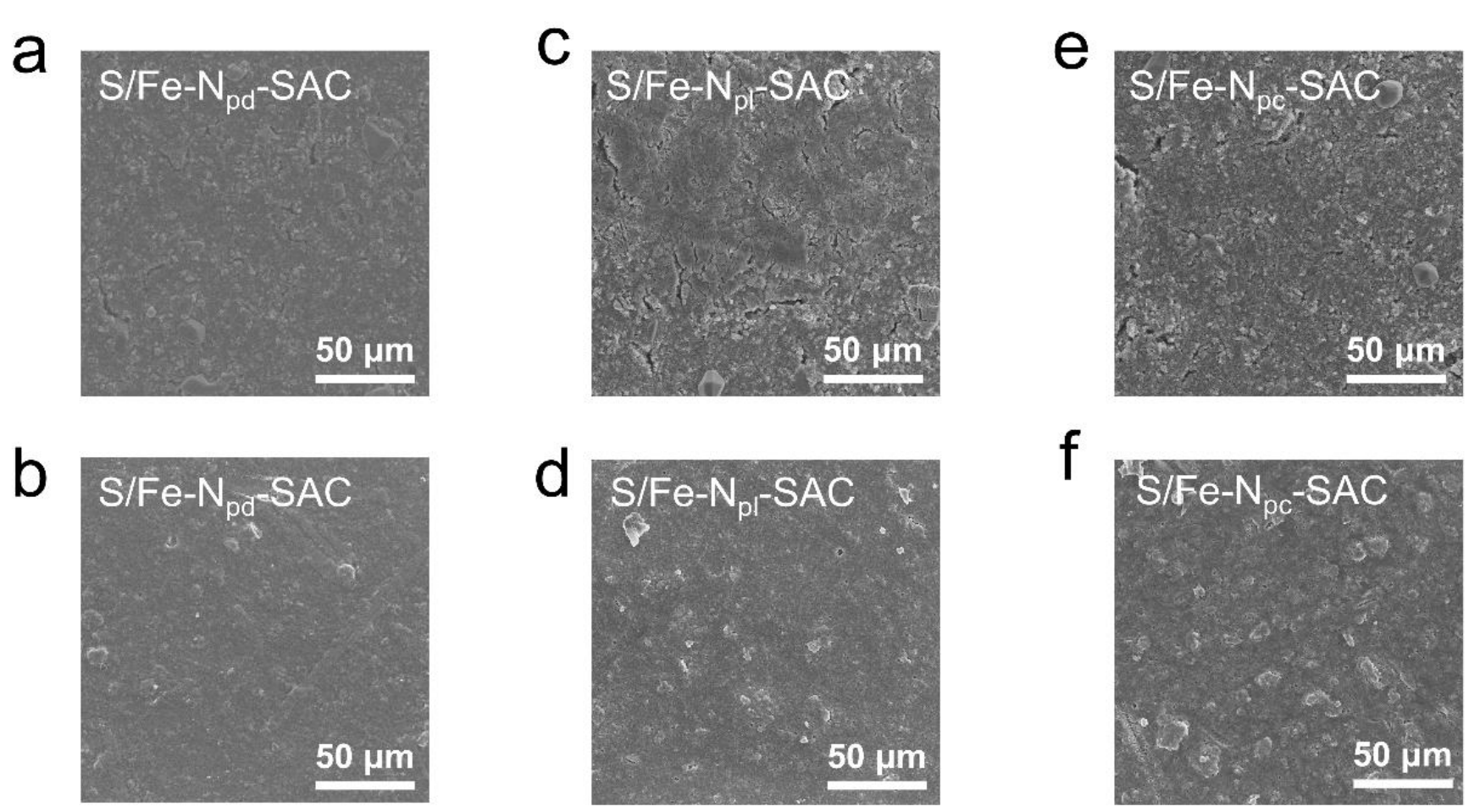

Figure S30. SEM images of the electrode surface after 100 cycles at 0.2 C: (a,b) S/Fe-N_{pd}-SAC, (c,d) S/Fe-N_{pl}-SAC and (e,f) S/Fe-N_{pc}-SAC.

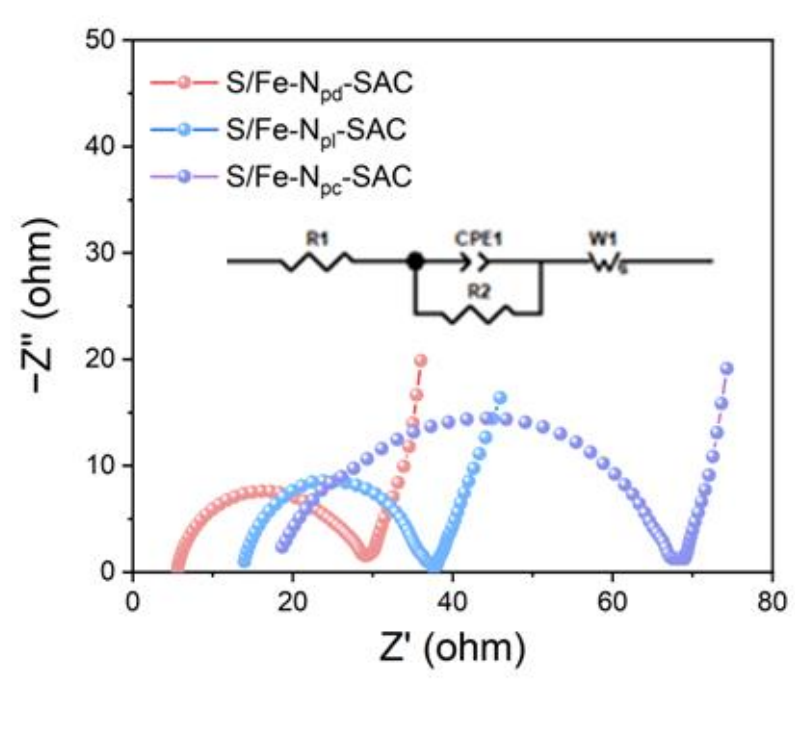

Figure S31. Nyquist plots of S/Fe-N_{pd}-SAC, S/Fe-N_{pl}-SAC and S/Fe-N_{pc}-SAC electrodes.

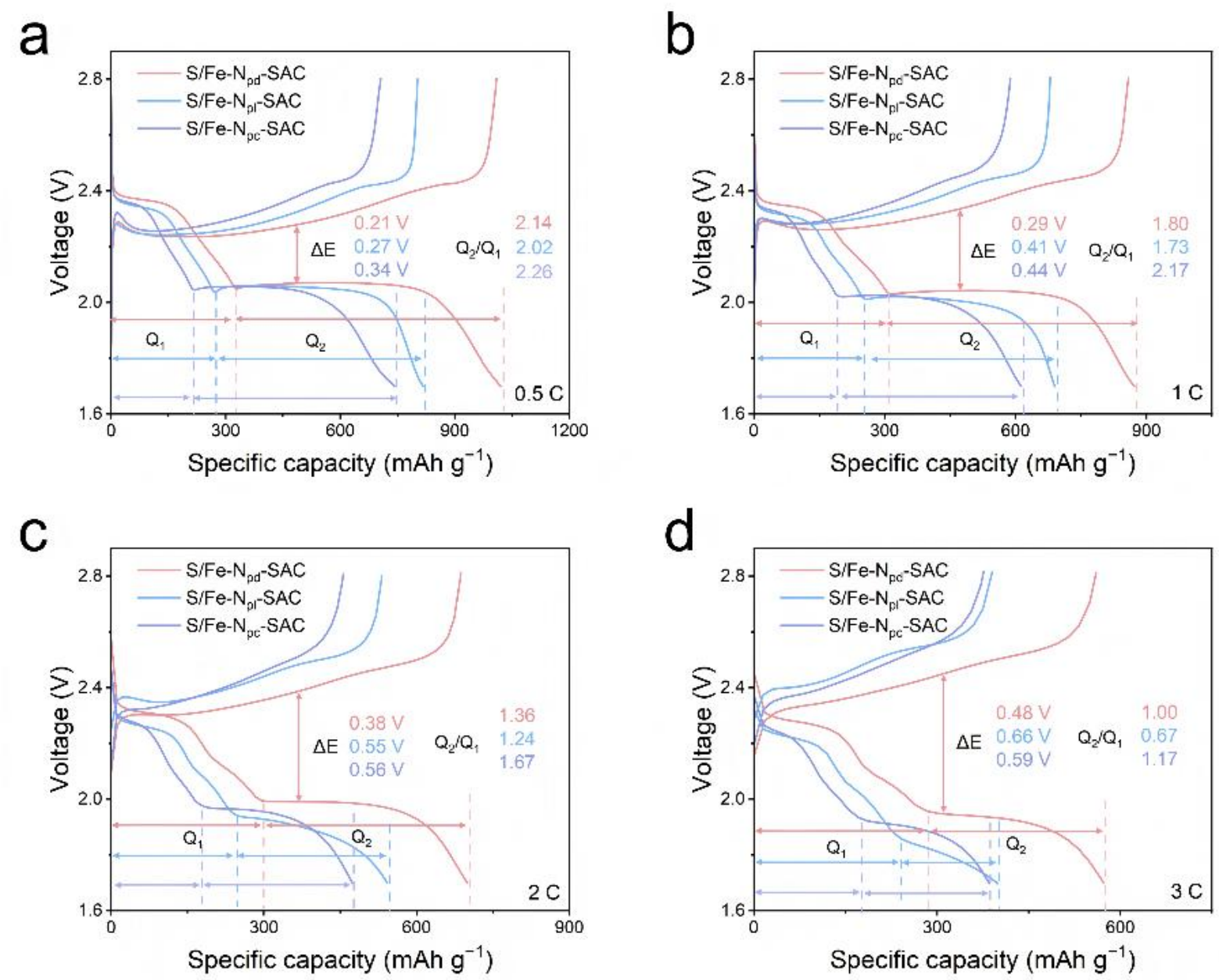

Figure S32. GCD profiles of S/Fe-N_{pd}-SAC, S/Fe-N_{pl}-SAC, and S/Fe-N_{pc}-SAC electrodes at varied rates.

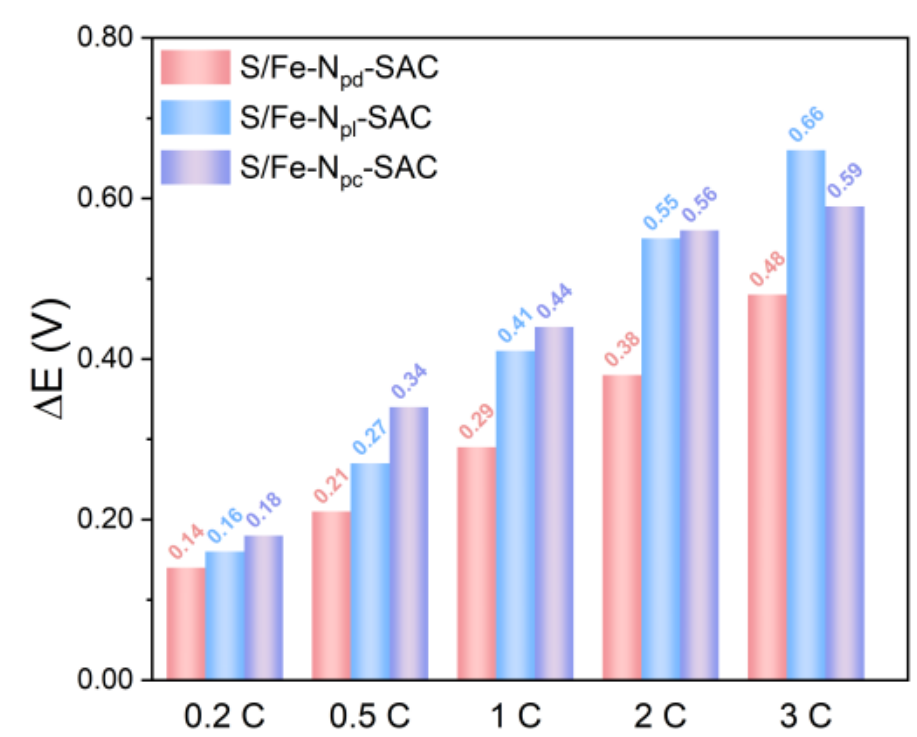

Figure S33. The voltage gap of S/Fe-N_{pd}-SAC, S/Fe-N_{pl}-SAC, S/Fe-N_{pc}-SAC electrodes at varied rates.

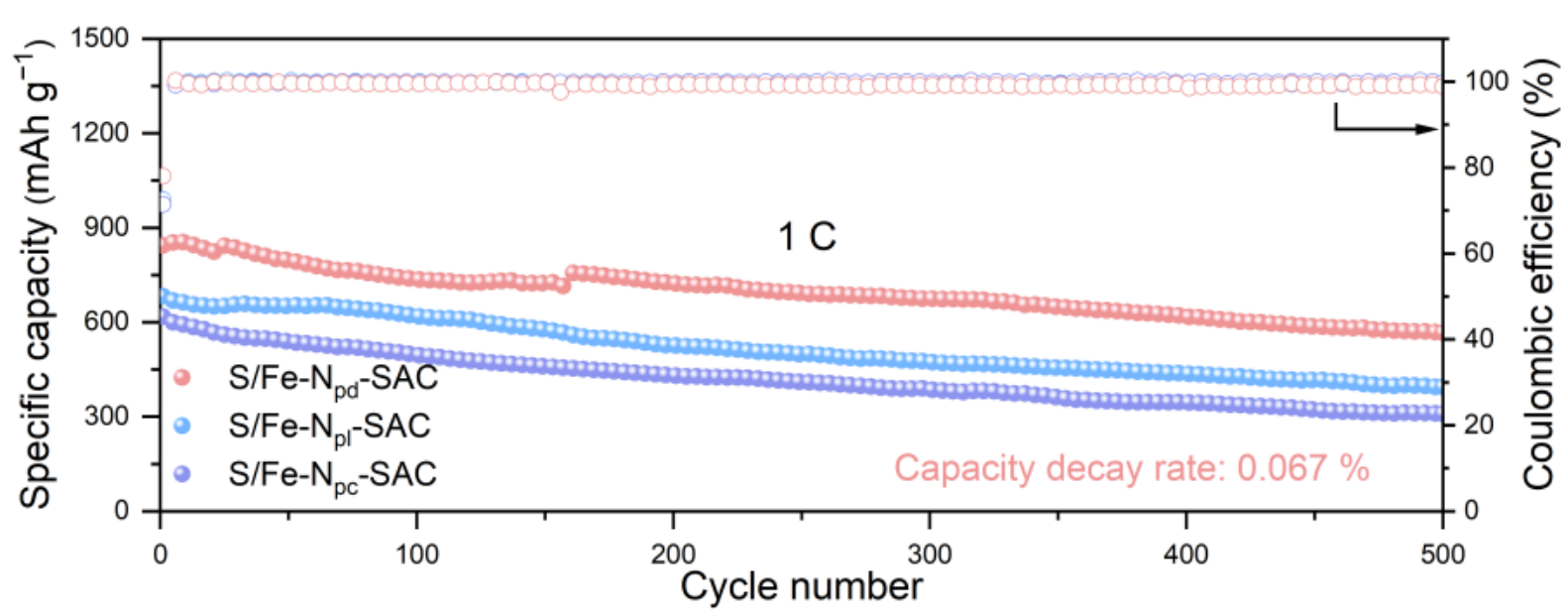

Figure S34. Cycling performances of S/Fe-N_{pd}-SAC, S/Fe-N_{pl}-SAC, S/Fe-N_{pc}-SAC electrodes at 1.0 C.

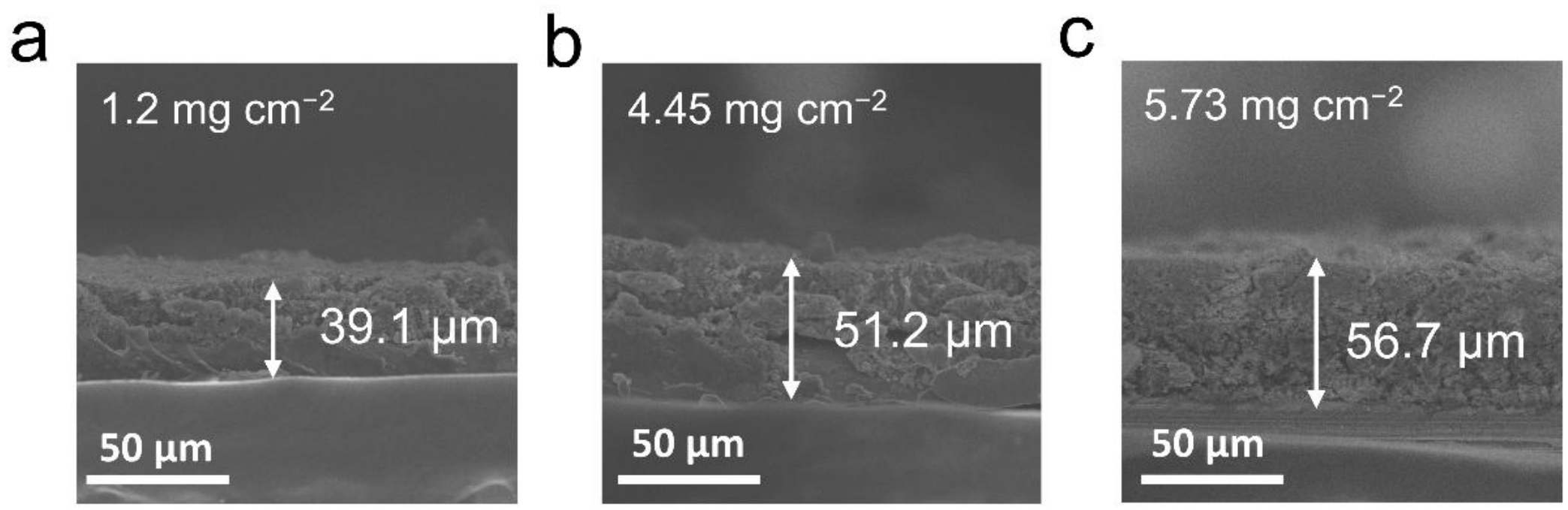

Figure S35. Cross-sectional SEM images of S/Fe-N_{pd}-SAC electrodes with different sulfur loadings.

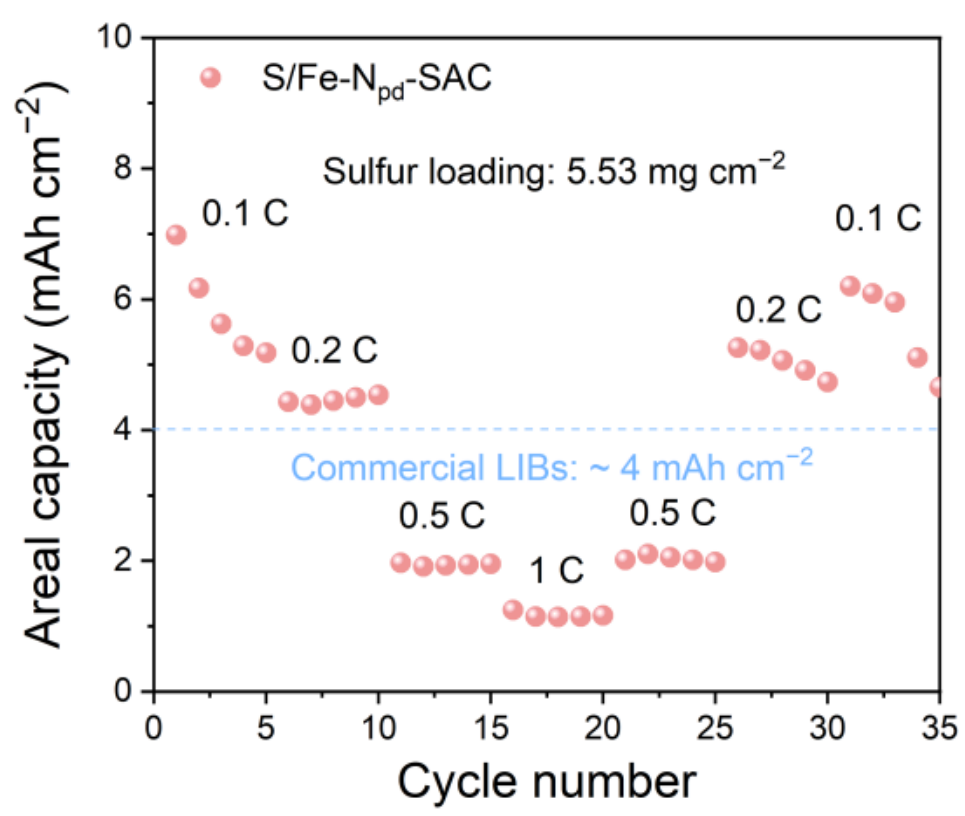

Figure S36. Rate performance of the S/Fe-N_{pd}-SAC electrode with a sulfur loading of 5.53 mg cm^{-2}.

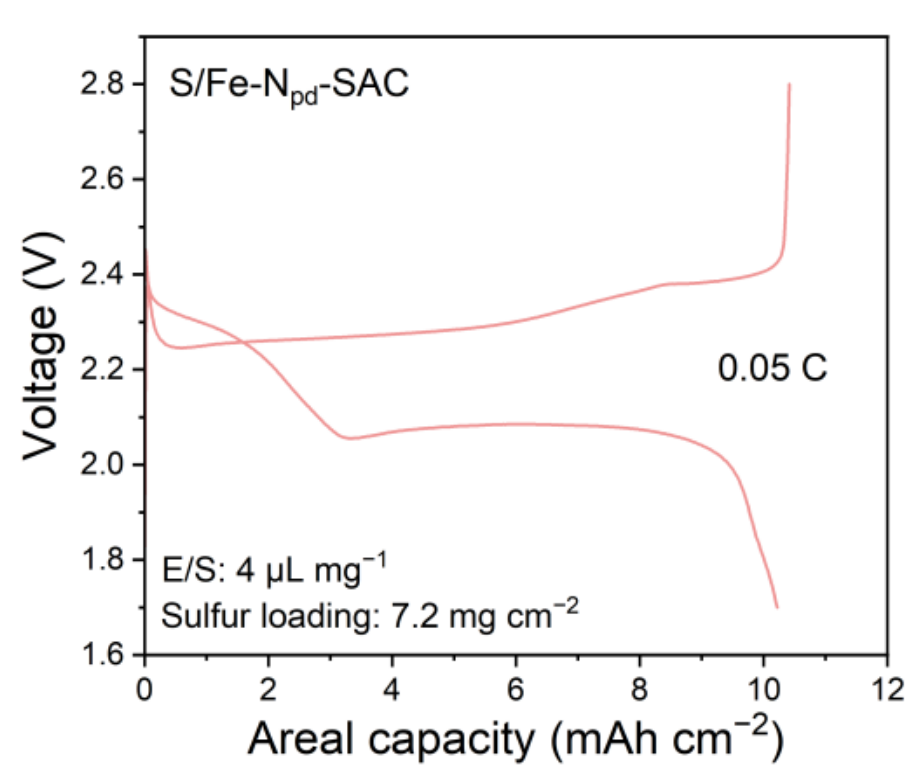

Figure S37. GCD profiles of S/Fe-N_{pd}-SAC electrode with a sulfur loading at 7.2 mg cm^{-2} and an E/S ratio 4 μL mg^{-1} at the first cycle (0.05 C).

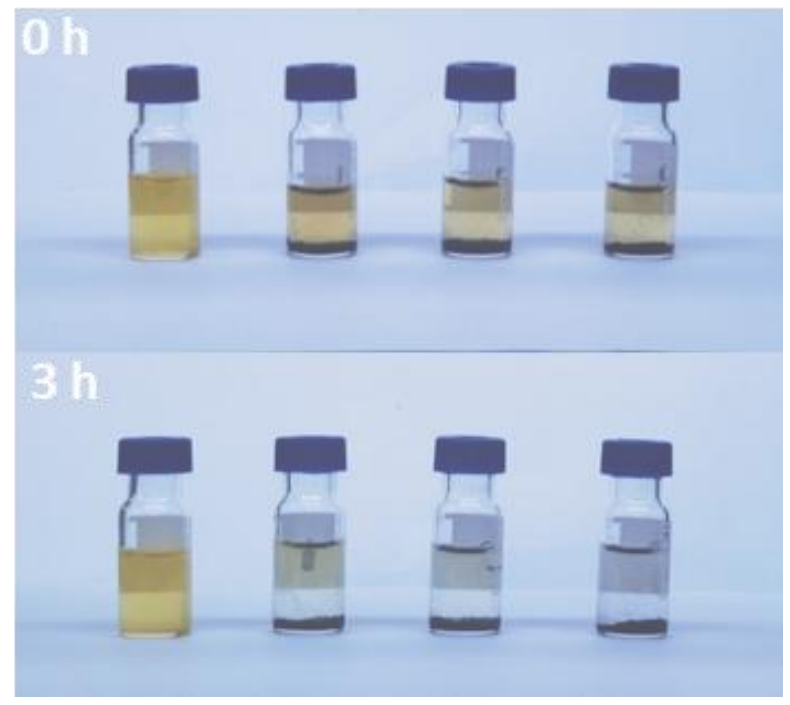

Figure S38. Digital photos of visualized adsorption tests in Li_2S_6 solution of bare Li_2S_6, Fe-N_{pc}-SAC, Fe-N_{pl}-SAC and Fe-N_{pd}-SAC, respectively.

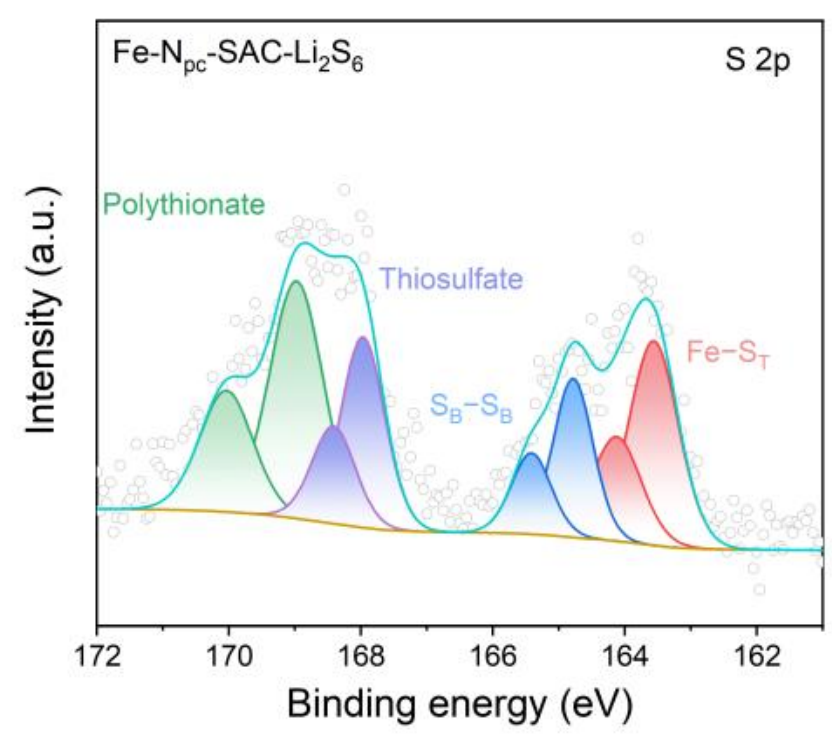

Figure S39. XPS S 2*p* spectrum of Fe-N_{pc}-SAC with Li_2S_6 after 3 h.

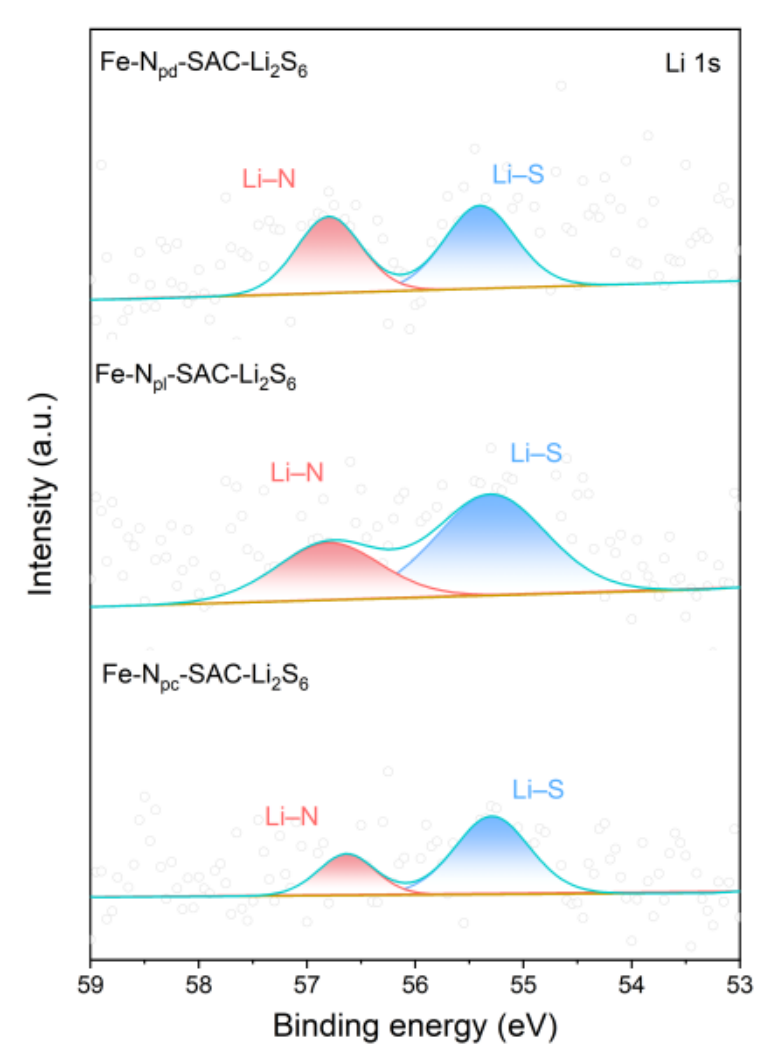

Figure S40. XPS Li 1*s* spectra of Fe-N_{pd}-SAC, Fe-N_{pl}-SAC or Fe-N_{pc}-SAC with Li_2S_6 after 3 h.

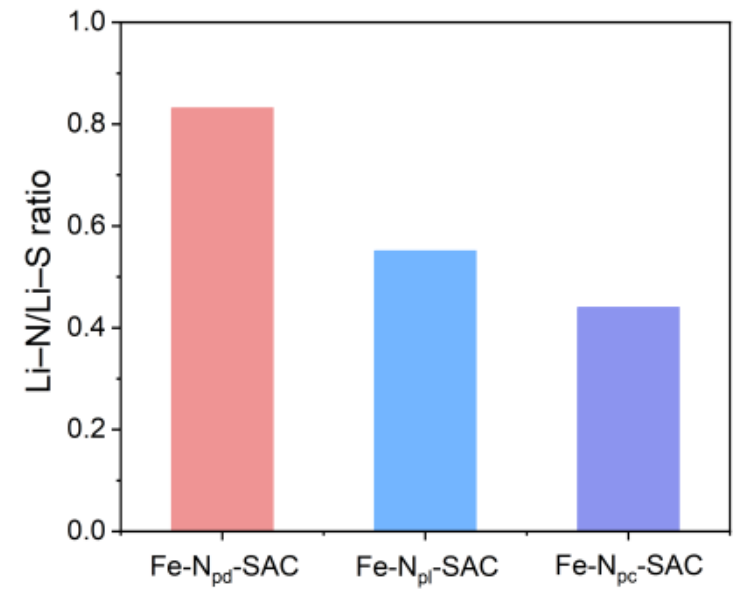

Figure S41. The Li–N/Li–S ratio value in Li 1*s* spectra of Fe-N$_{pd}$-SAC, Fe-N$_{pl}$-SAC and Fe-N$_{pc}$-SAC.

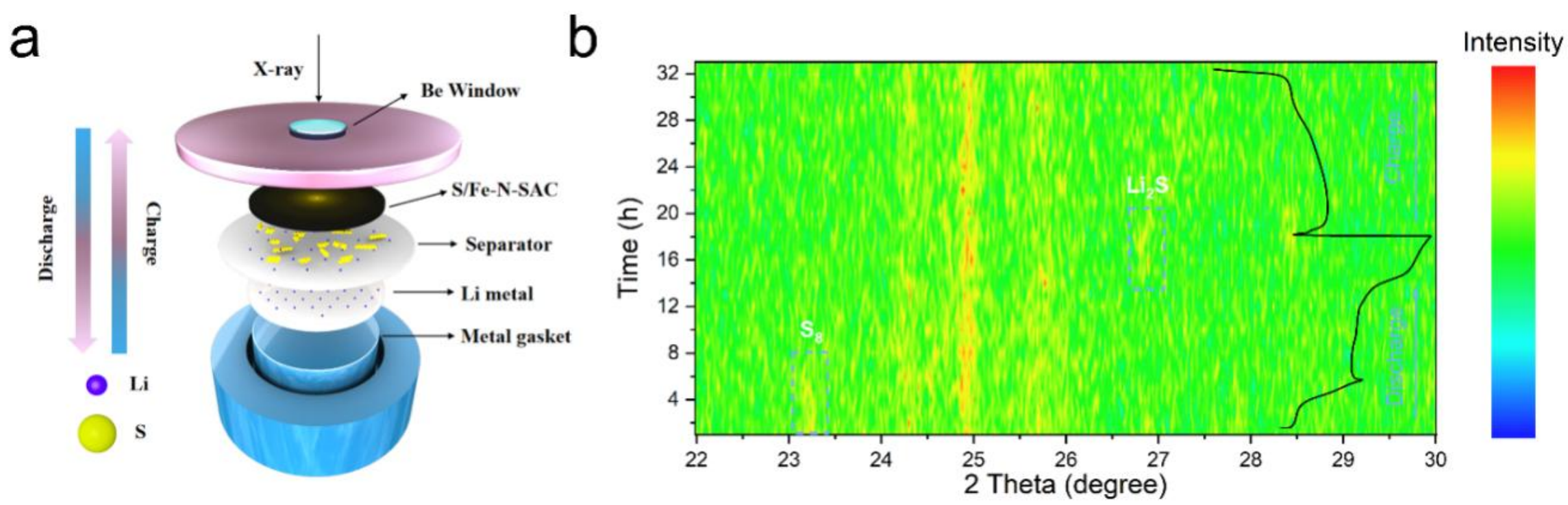

Figure S42. (a) Schematic illustration showing the Li–S cell set-up for *in situ* XRD test. (b)The *in situ* XRD contour maps for S/Fe-N$_{pd}$-SAC electrodes.

Supporting Table

Table S1. Fe element contents of Fe-N-SAC detected by ICP-OES.

Materials	ICP-OES (wt.%)
Fe-N_{pd}-SAC	0.353
Fe-N_{pl}-SAC	0.322
Fe-N_{pc}-SAC	0.272

Table S2. Comparison of nitrogen species content of three materials.

Materials	Pyridinic N (at. %)	Pyrrolic N (at. %)	Graphite N (at. %)
Fe-N_{pd}-SAC	32	26	42
Fe-N_{pl}-SAC	25	43	32
Fe-N_{pc}-SAC	25	22	53

Table S3. Comparison of electrochemical performances between Fe-N_{pd}-SAC and reported Fe SACs in Li–S batteries.

**Materials of Fe-based single-atom**	**Specific capacity (mAh g^{-1})**	**Capacity retention**	**Capacity decay rate**	**Ref.**
**Fe-N_{pd}-SAC**	**1265**	**95% (after 100 cycles at 0.2 C)**	**0.05% (after 500 cycles at 3.0 C)**	**This work**
channel-FeSAC	931	94.4%	0.093% (after 400 cycles at 1.0 C)	1
Fe-VO2@CB	1154	67% (after 100 cycles at 0.1 C)	0.066% (after 500 cycles at 1.0 C)	2
Fe-N5-C	1224	75.2% (after 100 cycles at 0.2 C)	0.054% (after 500 cycles at 1.0 C)	3
Fe@C-CNT	1164.4	53.5% (after 100 cycles at 0.1 C)	0.05% (after 200 cycles at 2.0 C)	4
FeNC-EEB-1	1011	80% (after 50 cycles at 0.05 C)	0.08% (after 250 cycles at 1.0 C)	5
FeCoNGr	878.7	77.4% (after 100 cycles at 0.2 C)	0.095% (after 500 cycles at 0.5 C)	6

Table S4. Comparison of sulfur loading conditions with Fe SACs in Li–S batteries.

Materials of Fe-based single-atom	S loading (mg cm^{-2})	Areal capacity (mAh cm^{-2})	Ref.
**Fe-N_{pd}-SAC**	**4.45**<br>**5.73**<br>**7.2**	**4.7**<br>**6.1**<br>**7.1**	**This work**
Fe-N5-C	4.3<br>6.3	4.4<br>5.4	3
FeNC-EEB-1	5	4.06	5
SA-Fe/Fe2N@NG	3<br>5	3.09<br>5.02	7
FeNC/wG	5.39	4.18	8
FeSA-PN@PNC	3.7<br>5	3.24<br>4.5	9
SA-Fe/VN@NMC	5.6	5.06	10

Supporting Reference

1. Zhu R, Zhao Z, Yan R *et al.* (2024). Sieve tube-inspired polysulfide cathode with long-range ordered channels and localized capture-catalysis microenvironments for efficient Li–S batteries. *Adv Funct Mater* 2024; **34**: 2314593.

2. Pang Y, Xie M, Lu X *et al.* (2024). Electronic metal-support interactions in atomically dispersed Fe(III)-VO_2 nanoribbons for high-performance lithium–sulfur batteries. *Adv Funct Mater* 2024; **34**: 2308849.

3. Zhang Y, Liu J, Wang J *et al.* (2021). Engineering oversaturated Fe-N_5 multifunctional catalytic sites for durable lithium-sulfur batteries. *Angew Chem Int Ed* 2021; **60**: 26622-26629.

4. Guo,J, Jiang H, Wang K *et al.* (2023). Enhancing electron conductivity and electron density of single atom based core–shell nanoboxes for high redox activity in lithium sulfur batteries. *Small* 2023; **19**: 2301849.

5. Lim W, Park C, Jung H *et al.* (2023). Cooperative electronic structure modulator of Fe single-atom electrocatalyst for high energy and long cycle Li–S pouch cell. *Adv Mater* 2023; **35**: 2208999.

6. Liu X, He Q, Liu J *et al.* (2023). Dual single-atom moieties anchored on N-doped multilayer graphene as a catalytic host for lithium–sulfur batteries. *ACS Appl Mater Interfaces* 2023; **15**: 9439-9446.

7. Ma C, Zhang Y, Feng Y *et al.* (2021). Engineering Fe–N coordination structures for fast redox conversion in lithium–sulfur batteries. *Adv Mater* 2021; **33**: 2100171.

8. Kim J, Kim S, Jung E *et al.* (2022). Atomic structure modification of Fe–N–C catalysts via morphology engineering of graphene for enhanced conversion kinetics of lithium–sulfur batteries. *Adv Funct Mater* 2022; **32**: 2110857.

9. Huang T, Sun Y, Wu J *et al.* (2022). Altering local chemistry of single-atom coordination boosts bidirectional polysulfide conversion of Li–S batteries. *Adv Funct Mater* 2022; **32**: 2203902.

10. Yang L, Wang X, Cheng X *et al.* (2023). Regulating Fe aggregation state via unique Fe–N–V pre-coordination to optimize the adsorption-catalysis effect in high-performance lithium-sulfur batteries. *Adv Funct Mater* 2023; **33**: 2303705.
